# The pathway-independent positive allosteric modulator C1 allows for the identification of active Y_4_ receptor relevant positions

**DOI:** 10.1007/s00018-025-06019-7

**Published:** 2026-01-13

**Authors:** Corinna Schüß, Oanh Vu, Tim Pelczyk, Mario Schubert, Yu Du, Jan Stichel, C. David Weaver, Jens Meiler, Annette G. Beck-Sickinger

**Affiliations:** 1 Institute of Biochemistry, Leipzig University, 04103 Leipzig, Germany; 2 Department of Chemistry, Vanderbilt University, Nashville, TN 37235 USA; 3 Institute of Pharmacology and Toxicology, Technische Universität Dresden, 01307 Dresden, Germany; 4 Department of Pharmacology, Vanderbilt University, Nashville, TN 37232 USA; 5 Institute of Chemical Biology, Vanderbilt University, Nashville, TN 37232 USA; 6 Institute for Drug Discovery, Leipzig University, 04103 Leipzig, Germany

**Keywords:** Neuropeptide Y_4_ receptor, Allostery, Pancreatic polypeptide, Mutagenesis, Receptor pharmacology, GPCR modulation

## Abstract

The neuropeptide Y_4_ receptor (Y_4_R) and its endogenous ligand pancreatic polypeptide (PP) are primarily involved in the regulation of satiety and energy balance and present relevant pharmacological targets. We characterized the novel Y_4_R positive allosteric modulator C1 that enhances Y_4_R G-protein signaling, ligand binding, and arrestin-3 recruitment to Y_4_R. Comparison with a close analog revealed the structural importance of an ethyl acetate moiety for Y_4_R affinity and PAM activity at the G-protein pathway. C1 shows a high selectivity for the Y_4_R, while signaling of the related subtypes Y_1_R, Y_2_R, and Y_5_R is not affected. Y_4_R G-protein signaling is even potentiated by the low-affinity agonists neuropeptide Y and peptide YY. Binding affinity of the endogenous ligands to Y_4_R is enhanced by C1, indicating a stabilization of the ligand-bound Y_4_R conformation. Using Y_4_R/Y_1_R chimera, important Y_4_R domains for C1 activity were identified. Single point mutagenesis and computational docking pinpointed hot-spot residues at Y_4_R important for stabilizing the active ligand-bound conformation.

**Supplementary Information:**

The online version contains supplementary material available at 10.1007/s00018-025-06019-7.

## Introduction

G protein-coupled receptors (GPCRs) represent a large and highly heterogeneous superfamily of cell surface receptors. They orchestrate numerous cellular functions in response to a versatile set of ligands [1]. GPCRs have emerged as major therapeutic targets and have become a central element in drug discovery in recent decades [2, 3]. Peptide-activated GPCRs are of particular interest as 32% of rhodopsin-like GPCRs naturally bind to peptides. Most drugs targeting peptide GPCRs that are either approved or under development are used for the treatment of metabolic diseases and cancer [4, 5]. Conventional GPCR-based drug discovery strategies have revolved around developing ligands based on the structure of native agonists or antagonists targeting the orthosteric site of a receptor [6], which are well characterized for many GPCRs, including the neuropeptide Y_4_ receptor (Y_4_R) [7]. In this study, we aim to understand receptor activation by characterizing a novel small-molecule ligand for Y_4_R. This allows us to broaden the pharmacological toolbox, complementing large peptide ligands, and opening new avenues in peptide GPCR drug discovery [6, 8–10]. Allosteric modulators of GPCRs have gained increasing attention, especially since three allosteric modulators, maraviroc, cinacalcet and, avacopan, have already made it to the clinic [11–14] and several more are under investigation in clinical trials [15]. Furthermore, advancements in high-throughput screening (HTS) technologies, structural elucidation, and computational methodologies will advance the rational and structure-based development of novel GPCR allosteric drugs within the near future [16, 17].

This study investigates the allosteric mechanism and binding pocket of a novel positive allosteric modulator (PAM) of the Y_4_R, termed C1 (VU0610218). The Y_4_R, a peptide GPCR mainly expressed in the gastrointestinal tract, regulates satiety and energy expenditure upon stimulation by its endogenous ligand pancreatic polypeptide (PP) [18–21]. Previous studies have demonstrated that activation of Y_4_R with selective agonists induces weight loss in mice [22–24] and reduces food intake in humans [25, 26]. Consequently, positive modulation of PP-mediated Y_4_R response emerges as a promising approach to augment the physiological signaling, and advance the Y_4_R as clinically relevant GPCR. Previously, niclosamide and tBPC have been identified as Y_4_R positive allosteric modulators, however, these compounds exhibit limited selectivity or show agonistic activity at Y_4_R in the absence of the endogenous peptide ligands [27, 28]. In this study, C1 was identified as structurally novel Y_4_R pure PAM in a Ca^2+^ flux-based high-throughput screening (HTS). The Y_4_R is part of a multiligand/multireceptor system comprising the closely related receptors Y_1_R, Y_2_R, and Y_5_R and the peptide ligands PP, peptide YY (PYY) and neuropeptide Y (NPY) [29]. Therefore, the effect of the small molecule C1 and its analog C2 was also investigated for Y receptor selectivity and functional ligand selectivity. Using different functional assay set-ups, we aim to understand the molecular mechanism of this small molecule at Y_4_R with respect to G-protein signaling and binding. As Y_4_R promiscuously couples to different intracellular signaling proteins, the effect of C1 on arrestin recruitment to Y_4_R is considered in addition to the G-protein pathway. By mutagenesis approaches using Y_4_R/Y_1_R chimeras and single mutants, as well as computational docking using the recently described Y_4_R-PP-G_i1_ cryo-electron microscopy (cryo-EM) structure [7], we present the allosteric binding pocket of C1 at the active state of Y_4_R.

## Materials and methods

### Material

#### Molecular biology and functional assays

Dulbecco’s Modified Eagle’s Medium (DMEM), Hank’s Balanced Salt Solution (HBSS), Dulbecco’s Phosphate-Buffered Saline (DPBS), trypsin-EDTA, and Ham’s F12 were obtained from Lonza (Basel, Switzerland). DMEM/F-12 medium without phenol red was purchased from Gibco™, Thermo Fisher (Waltham, MA, USA). G418 sulfate and hygromycin B were from Invivogen (Toulouse, France). Fetal bovine serum (FBS) was purchased from Biochrom GmbH (Berlin, Germany). OptiMEM was received from Life Technologies (Basel, Switzerland). Dimethylsulfoxide (DMSO), Pluronic F-127, HEPES, Hoechst33342, probenecid, forskolin, TRIS, polyethylenimine, Pefabloc, calium chloride (CaCl_2_) and magnesium chloride (MgCl_2_) were purchased from Sigma-Aldrich (St. Louis, MO, USA). Bovine serum albumin (BSA) was obtained from Carl Roth (Karlsruhe, Germany). MetafectenePro transfection reagent was acquired from Biontex Laboratories GmbH (München, Germany). Lipofectamine™ 2000 transfection reagent and competent *Escherichia coli* (*E.coli*) DH5α were purchased from Invitrogen (Carlsbad, CA, USA). Coelenterazine h was received from NanoLight Technology (Pinetop, AZ, USA). Fluo-2 AM was from Abcam (Cambridge, UK). GloSensor cAMP reagent was obtained from Promega (Fitchburg, WI, USA). Human ^125^I–PP and ^125^I–PYY were purchased from PerkinElmer (Waltham, MA, USA). *DpnI*, deoxyribonucleotide (dNTP) mix, Phusion High-Fidelity Polymerase, and 5× HF buffer were obtained from Thermo Fisher Scientific (Waltham, MA, USA). Sense and antisense primers were purchased from Biomers (Ulm, Germany). Wizard Plus Mini and Midi DNA purification kits were purchased from Promega (Fitchburg, WI, USA).

#### Compounds

C1 (VU0610218, product ID: F3315-0069) and C2 (product ID: F3315-0071) were ordered from Life Chemicals (Kyiv, Ukraine). All compounds are > 95% pure by HPLC (Fig. S6).

### Plasmids

For signaling studies, the coding sequence of human Y_4_R, C-terminally fused to eYFP, was cloned into the mammalian expression vector pEYFP_N1 (Clontech, Heidelberg, Germany), and pVitro2-hygro-mcs vector (Invivogen), as described in earlier studies [30, 31]. The chimeric G protein ∆6Gα_qi4−myr_ was kindly provided by Evi Kostenis (Rheinische Friedrich-Wilhelms-Universität, Bonn, Germany) [32]. Arrestin BRET was performed with bovine arrestin-3, N-terminally tagged with Venus fluorophore, and Y_4_R, C-terminally fused to *Renilla* luciferase 8 and cloned into the pcDNA3 vector [33, 34]. The pGloSensor-22 F cAMP plasmid (Cat. # E2301) was obtained from Promega (Fitchburg, WI, USA). The G protein sensors (G_o1_-CASE RRID: Addgene_168123, G_i3_-CASE RRID: Addgene_168122) were kindly provided by Hannes Schihada and Gunnar Schulte (Karolinska Institute, Stockholm, Sweden).

### Mutagenesis

Single amino acid substitutions or segments of 2–4 adjacent residues were introduced into wildtype Y_4_R-eYFP_N1 plasmid by site-directed mutagenesis PCR using Phusion High-Fidelity Polymerase and 5× HF buffer according to the manufacturer’s protocol. Sense and antisense primers were used at a final concentration of 0.5 µM, and dNTP mix was added at a final concentration of 200 µM. *DpnI* was used to digest parental methylated DNA for 3 h at 37 °C. PureYield Plasmid MiniPrep and MidiPrep DNA Purification Kits were used to isolate plasmid DNA after transformation of the cDNA into competent *E.coli* DH5α, as recently described [35]. The sequence of all Y_4_R mutant constructs was confirmed by Sanger dideoxy sequencing. Membrane expression and signaling of Y_4_R variants was investigated by live cell microscopy and are summarized in Fig. S3 and [35].

### Cell culture

All cell lines were cultivated in 75 cm^2^ cell culture flasks in a humidified atmosphere at 37 °C and 5% CO_2_, and passaged twice a week. The stable cell lines COS-7_hY_1/2/4/5_R-eYFP_∆6Gα_qi4−myr_, co-expressing either Y_1_R, Y_2_R, Y_4_R, or Y_5_R, C-terminally fused to eYFP fluorophore, and the chimeric G protein ∆6Gα_qi4−myr_ were maintained in DMEM supplemented with 10% heat-inactivated FBS, 133 µg/mL hygromycin B, and 1.5 mg/mL G418 sulfate. Wildtype COS-7 cells were obtained from the American Type Culture Collection (ATCC, Manassas, VA, USA; CRL-1651, RRID: CVCL_0224ATCC) and cultured in DMEM supplemented with 10% heat-inactivated FBS. HEK293 cells, obtained from the Deutsche Sammlung von Mikroorganismen und Zellkulturen (DSMZ, Braunschweig, Germany; ACC-305, RRID: CVCL_0045), were cultured in DMEM/Ham’s F12 medium (1:1 *v/v*) supplemented with 15% heat-inactivated FBS. Stably transfected HEK293-hY_4_R-eYFP cells were maintained in DMEM/Ham’s F12 medium (1:1 *v/v)*, 15% heat-inactivated FBS, and 100 µg/mL hygromycin. All cell lines were routinely tested negative for mycoplasma contamination.

### Ca^2+^ flux assay

Ca^2+^ flux assays were performed in COS-7 cells stably co-expressing hY_4_R-eYFP and the G protein chimera ∆6Gα_qi4−myr_, as previously described [35]. Compound selectivity studies were performed in stably transfected COS-7_hY_1/2/5_R-eYFP_∆6Gα_qi4−myr_ cells. For mutagenesis studies, COS-7 cells were transiently co-transfected with Y_4_R-eYFP WT or mutant plasmid, and the chimeric G protein Δ6Gα_qi4−myr_ (ratio 3:1) overnight using Metafectene Pro transfection reagent, according to the manufacturer’s protocol. Transiently or stably transfected COS-7 cells were seeded in black clear bottom 96-well plates (Greiner, Kremsmünster, Austria, #655090) at a density of 30,000 cells/well and incubated for at least at 24 h at 37 °C. Ca^2+^ flux assay was performed as described previously [27, 35]. In brief, cell culture medium was removed, cells were labeled with 100 µL/well of Fluo-2 AM dye solution (2.4 µM Fluo-2 AM, 0.06% (*v/v*) Pluronic-F127) in assay buffer (20 mM HEPES, 2.5 mM probenecid in HBSS, pH 7.5) and incubated for 60 min at 37 °C. Afterwards, the dye solution was replaced by 50 µL/well assay buffer and basal Ca^2+^ levels were detected for 20 s with a FlexStation III device (Molecular Devices, San Jose, CA, USA) using a fluorescent read-out (λ_ex_ = 485 nm, λ_em_ = 525 nm). After 20 s, 50 µL/well of DMSO control or 2× compound solution was automatically added and Ca^2+^ response was measured for 60 s. Following, 20 µL/well of 6× serially diluted ligand solution (for full curves) or a single PP EC_30_ concentration (for mutagenesis studies) was added. Ca^2+^ response was measured over a total run time of 140 s. Raw data were quantified as *x*-fold over basal. Normalization and calculation of nonlinear regression were performed with GraphPad Prism 9.0 (GraphPad Software, San Diego, CA, USA; RRID: SCR_002798). Allosteric parameters were determined by using an operational model of allosterism (1) [36], whereas *E* describes the pharmacological effect, *A* and *B* are the concentration of the orthosteric and allosteric ligand, respectively, *K*_*B*_ and *τ*_*B*_ represent the equilibrium dissociation constant and the operational index of efficacy of the allosteric modulator, *EC*_*50*_ is the half-maximal molar concentration of the orthosteric ligand, *αβ* is referred to as cooperativity factor, and *E*_*m*_ and *n* represent the maximal responsiveness of the system and the Hill slope, respectively.

1 $$\:E=\:Basal+\frac{\left({E}_{m}-Basal\:\right){\left(\left[A\right]\left({K}_{B}+\alpha\:\beta\:\left[B\right]\right)+{\tau\:}_{B}\left[B\right]\left[E{C}_{50}\right]\right)}^{n}}{{{[EC}_{50}]}^{n}({K}_{B}+\left[B\right]{)}^{n}{+\left(\left[A\right]\left({K}_{B}+\alpha\:\beta\:\left[B\right]\right)+{\tau\:}_{B}\left[B\right]\left[E{C}_{50}\right]\right)}^{n}}$$

### G protein sensor BRET

Direct G-protein activation was investigated in a BRET-based approach using G protein sensors [37]. Plasmids encoding native Y_4_R (500 ng) and tricistronic G protein biosensor (500 ng) were co-transfected into 80% confluent COS-7 cells in 25 cm^2^ cell culture flasks using Metafectene Pro transfection reagent, according to the manufacturer’s protocol. To ensure Y_4_R-specific G-protein activity, mock vector (pcDNA3 plasmid) was used as control instead of Y_4_R plasmid. The day post transfection, cells were resuspended in phenol red-free DMEM/F-12 supplemented with 10% FBS, and reseeded into white solid 96-well plates at a density of 50,000 cells/well for another 24 h. Prior to BRET measurements, cell medium was replaced by 50 µL of BRET buffer (25 mM HEPES in HBSS, pH 7.4). After the addition of 50 µL of coelenterazine h (4.2 µM final concentration), cells were incubated for 5 min at 37 °C, following the addition of 50 µL of 4× concentrated compound solution (0.3–30 µM) for another 3 min at 37 °C. For endpoint measurements, cells were stimulated with 50 µL of varying PP concentrations (10^− 6^ to 10^− 12^ M final concentration) or buffer as control, and incubated for 20 min at 37 °C. Fluorescence (400–470 nm) and luminescence (535–600 nm) were measured well-wise with an integration time of 500 ms using a Tecan Spark plate reader (Tecan, Männerdorf, Switzerland). For kinetic measurements, fluorescence and luminescence were continuously recorded for 35 min at 37 °C in live COS-7 cells. After the addition of coelenterazine h and compound solution at different concentrations, the plate was placed in the plate reader and baseline was detected for 5 min, followed by the stimulation of the cells with 1 nM of PP final concentration. Raw BRET was calculated by dividing the fluorescence values by the luminescence values. NetBRET values were obtained by subtraction of the BRET values of the buffer control from stimulated values. For kinetic measurements, the netBRET values from the buffer control were additionally subtracted from the stimulated values at each time point of the measurement.

### Live cell glosensor cAMP measurement

The activation of the endogenous Y_4_R G_i/o_ pathway in response to PP was monitored using Glosensor cAMP assay (Promega) in COS-7 cells, according to the manufacturer’s protocol. In brief, COS-7 cells at a density of 70–80% were transiently co-transfected with Y_1/2/4/5_R-eYFP and pGloSensor-22 F cAMP plasmid (ratio 1:1, endpoint measurement) or Y_4_R-eYFP and the pGloSensor-22 F cAMP plasmid (ratio 1:1, kinetic measurement) overnight using Metafectene Pro transfection reagent. The following day, the cells were reseeded into white solid 384-well plates with a density of 15,000 cells (endpoint measurements) or white solid 96-well plates at a density of 50,000 cells per well (kinetic measurement) and incubated for 24 h under standard cell culture conditions. On the assay day, cell culture medium was aspirated and cells were equilibrated with 20 µL/well (endpoint measurements) or 100 µL/well (kinetic measurement) of a 2% (*v/v*) GloSensor cAMP reagent stock solution in buffer (HBSS with 25 mM HEPES) for 2 h at RT until a stable basal signal was obtained. For endpoint measurement, compound solutions (0.3, 1, 3, 5, 10, 20, and 30 µM of C1 final concentration), peptide dilutions (ranging from 1 pM to 10 µM final concentration) and forskolin (5 µM final concentration) were prepared in BRET buffer at RT. Following incubation, cells were treated with 5 µL/well of 8× compound solution or DMSO as control. After an incubation of 5 min, 5 µL/well of 8x peptide solution or buffer was added, and cells were incubated for additional 5 min. Subsequently, 10 µL/well of 4x forskolin solution was added. The luminescence signal was measured 20 min after addition of forskolin with an integration time of 500 ms using Tecan Spark plate reader. A non-linear regression was performed using GraphPad Prism 10.6.1 and curves were normalized to DMSO control. For kinetic measurement, compound solutions (0.3, 3, and 30 µM of C1 final concentration), peptide dilution (PP at a final concentration of 1 nM) and forskolin (10 µM final concentration) were prepared in BRET buffer at RT. Following incubation, cells were treated with 25 µL/well of 8× compound solution or DMSO as control. Subsequently, the plate was placed in a Tecan Spark plate reader and luminescence was recorded continuously over 45 min with an integration time of 500 ms. After 5 min, the run was paused, cells were stimulated with 50 µL/well of the agonist PP (1 nM, 4× concentrated), and after 10 min forskolin, a direct activator of the adenylyl cyclase, at a final concentration of 10 µM was added. For data evaluation, baseline values (only compound solution in buffer) were removed from each value. Data were normalized to the maximal DMSO control (100%) and baseline control (0%). GraphPad Prism 9.0 was used to plot luminescence signals over time.

### Y_4_R membrane preparation

Y_4_R membranes were obtained from stably transfected HEK293_Y_4_R-eYFP cells. At a confluency of about 90%, cells were harvested using DPBS and collected by centrifugation for 5 min at 1,800 rpm, 4 °C. The next steps were continuously performed on ice. The cell pellet was resuspended in ice-cold tris buffer (50 mM tris, 50 µM Pefabloc, pH 7.5) and homogenized using a Dounce homogenizer. The cell suspension was centrifuged for 20 min at 2,400 rpm, 4 °C. The obtained supernatant was carefully collected and centrifuged for 60 min at 12,000 rpm, 4 °C. The supernatant was discarded and the pellet containing membranes was resuspended in ice-cold HEPES buffer (25 mM HEPES, 25 mM CaCl_2_, 1 mM MgCl_2_, 50 µM Pefabloc, pH 7.4) and homogenized with a Dounce homogenizer. The suspension was again centrifuged for 60 min at 12,000 rpm, 4 °C. The supernatant solution was discarded, and the pellet was suspended in HEPES buffer. Protein concentration was determined using Bradford protein assay [38]. Aliquots of membrane preparations were stored at − 80 °C until use.

### Radioligand binding assay

Radioligand binding experiments were performed using membrane preparations of stably transfected HEK293-hY_4_R-eYFP cells, as described [27, 35]. In short, DMSO vehicle and C1 solutions were prepared in ice-cold HEPES buffer (25 mM HEPES, 2.5 mM CaCl_2_, 1.0 mM MgCl_2_, 1% BSA, 1% 5 mM Pefabloc) and 40 µL/well were added to a translucent non-binding 96-well plate (Greiner, #655901). Y_4_R membrane preparations were gently thawed on ice, suspended in cold HEPES buffer, and added to the DMSO and C1 solutions (40 µL/well, final concentration 0.3 µg). ^125^I-PP (human) or ^125^I-PYY (human) peptide solutions were prepared in MilliQ water, supplemented with 1.0% BSA. Ten µL of 10× radiolabeld peptide solutions (30 pM ^125^I-PP or 150 pM ^125^I-PYY, final concentration) were added to Y_4_R membranes. To determine unspecific binding, 10 µL of unlabled PP at a final concnetration of 1 µM was added instead of 10 µL of 1.0% BSA. Binding assays were incubated for 4 h at RT under gentle shaking until equilibrium has reached. Afterwards, Y_4_R membrane-bound ^125^I-PP or ^125^I-PYY were separated by filtration with a GFC filter (PerkinElmer), presoaked with 0.1% polyethylenimine (*w/v*) in PBS using a MicroBeta Filtermat-96 Cell Harvester System (PerkinElmer). Membranes were washed three times with ice-cold PBS, dried for 15 min at 55 °C, and treated with MeltiLex solid scintillation sheets. Radioactivity of Y_4_R-bound ^125^I-PP or ^125^I-PYY was measured using a MicroBeta 2 scintillation counter (PerkinElmer).

### Arrestin BRET

Arrestin-3 recruitment to Y_4_R was investigated by BRET assay in transiently transfected COS-7 cells, as described [27]. For transient transfection, COS-7 cells were grown in 75 cm^2^ cell culture flasks to a density of about 80% and transfected with 1,000 ng Y_4_R-Rluc8 and 11,000 ng Venus-arrestin-3 using Metafectene Pro, according to the manufacturer’s protocol. The next day, cells were resuspended in phenol red-free DMEM/F-12 supplemented with 10% FBS, and seeded into white solid 96-well plates (Greiner, #655083) at a density of 50,000 cells/well. COS-7 cells were incubated for at least 24 h under standard conditions. For BRET measurements, cell medium was removed and 100 µL of BRET buffer (HBSS, 25 mM HEPES, pH 7.4) containing DMSO vehicle or a final concentration of 30 µM of C1 was added. For endpoint measurements, 50 µL of 4× serially diluted peptide ligand was added, followed by the addition of 50 µL of coelenterazine h at a final concentration of 4.2 µM. The plate was incubated for 5 min at 37 °C before measurement was performed with a Tecan Infinite M 200 Plate Reader (Tecan) using the filter sets GREEN1 (Venus fluorophore, 520–570 nm) and BLUE1 (Rluc8, 370–480 nm). For kinetic studies, 50 µL of coelenterazine h (4.2 µM final concentration) was added, and baseline was measured for 5 min in the plate reader. Afterwards, cells were subsequently stimulated with agonist or blank (BRET buffer), and BRET signals were measured continuously over 30 min at 37 °C in a Tecan Infinite M 200 Plate Reader. BRET signals were calculated as the ratio of fluorescence divided by luminescence. netBRET signals were obtained by the subtraction of the BRET ratio of unstimulated cells from stimulated cells.

### Data analysis and statistics

Calculations of mean, SEM, and nonlinear regression were performed using the statistics program GraphPad Prism 9.0 and 10 (GraphPad Software, San Diego, CA, USA; RRID: SCR_002798). For statistical analysis, unpaired t-test, or one-way ANOVA with Dunnett’s multiple comparison post test was applied. Data are expressed as mean ± SEM. The applied statistical tests and exact sample sizes are given in the respective Figure legends.

### Optimization of the PP-Y_4_R cryo-EM structure

The PP-Y_4_R optimized complex structure were generated as described previously [39]. Only the atom coordinates of the PP-Y_4_R complex were extracted from the cryo-EM structure (PDB ID: 7X9C) [7]. The atom names of the N-terminal amidated tyrosine residue of PP was manually changed to match with Rosetta’s naming convention. The PP-Y_4_R complex structure was then relaxed with constraints to the starting structures [40]. The 100 output relaxed models were used as templates for the FlexPepDock refinement peptide docking protocol [41] to generate 3,000 output models. The top 30 docking models with the best interface interaction scores were selected to be the templates for the next step of ligand docking.

### Induced-fit docking of C1 to the optimized of PP-Y_4_R cryo-EM structure

A set of 100 conformations of C1 was generated using the ConformerGenerator application of BCL::Conf [42–44]. This application builds small-molecule conformations from substructures derived from small molecule crystal structures in the Crystallography Open Database (COD; RRID: SCR_005874). RosettaLigand (Rosetta 3.12; RRID: SCR_015701) [45, 46] was used to dock C1 to PP-Y_4_R models. A starting position was selected for C1 based on the proximity to residues that are important to C1 activity according to mutagenesis data. The induced-fit docking protocol started with an initial docking round that allows the ligands conformation sampling as well as rotation and translation. The docking protocol included a low resolution (centroid mode) phase consisting of 500 cycles sampling ligand conformers in 4 Å translation search and complete reorientation search, and a high-resolution phase consisting of six cycles of sidechain refinement with small perturbations of ligand poses and conformation. Throughout the conformation sampling process, constraints were set so that the ligand would not move too far from the initial placement. Then, another round of relaxing the backbone of the residues surrounding the ligand to mimic the induced fit effect, and a final refinement docking to optimize the ligand-receptor atomic interactions was performed [40]. During the refinement phase, the translation search was reduced to 1 Å. This phase finds an energetically favorable pose by combining minor ligand conformational flexibility with sidechain refinement simultaneously. The top 5% output models by interface delta score were collected, then clustered into 3 clusters. The Rosetta interface scores versus ligand RMSDs graphs after the final round of induced fit docking are shown in Fig. S7.

### Mutagenesis agreement score computation on output docking clusters

For each of the three clusters, the mutagenesis agreement score was computed such that the scores are higher when an important residue (according to mutagenesis data) has favorable binding energy toward the ligand. The mutagenesis agreement score of a cluster t is calculated as mutagenesis agreement score (t) = - ∑PRIEi, where PRIEi is the predicted per-residue interaction energy between the ligand and an important residue. Mutagenesis agreement scores for all three clusters are listed in the Supplementary Table S2.

### *Gain-of-function* Y_4_R mutant prediction

For each cluster, the top 10 models were used as template models for the calculations. The residues that interact with C1 were redesigned with the Rosetta binding pocket design protocol [47]. Only one residue was allowed to mutate during each run. Only the output Y_4_R mutants that showed improvement in interface scores were selected.

## Results

### Identification of C1 as Y_4_R PAM at the G-protein signaling pathway

**Fig. 1:**
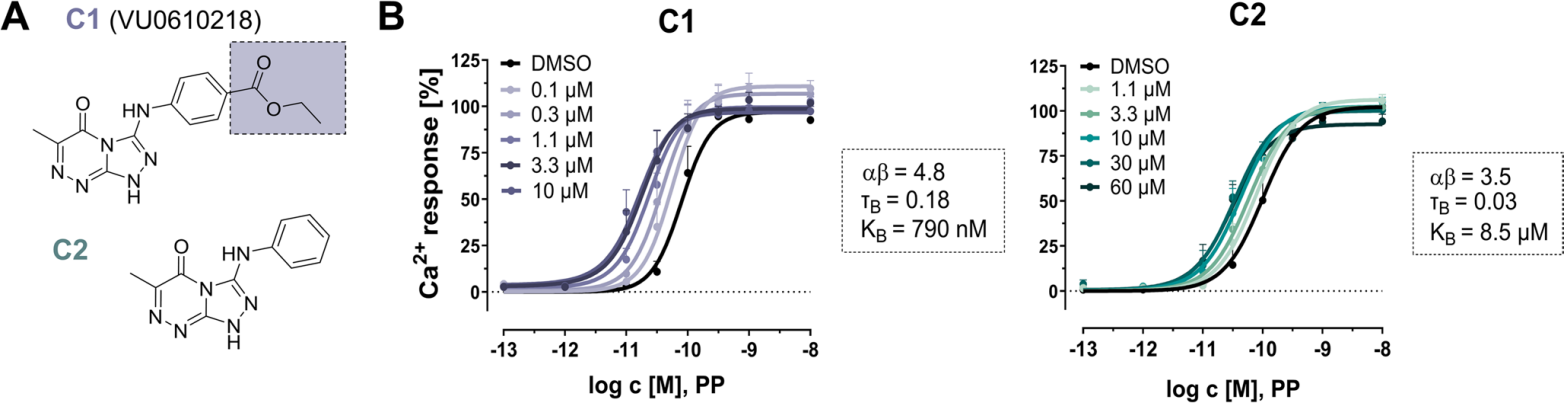
C1 and C2 potentiate Y_4_R G-protein signaling studied by Ca^2+^ flux assay. (**A**) Structure of C1 and C2 with the additional ethyl acetate moiety of C1 highlighted in lilac. (**B**) Titration of increasing concentrations of C1 and C2 induce a concentration-dependent leftward-shift of PP concentration-response curves compared to the DMSO control. Data were obtained by Ca^2+^ flux assay in COS-7_hY_4_R-eYFP_∆6Gα_qi4-myr_ cells. The effects of C1 (> 1 µM) and C2 (> 10 µM) are saturable, indicating allosteric behavior. Data were fitted to an operational model of allosterism to calculate allosteric parameters: K_B_ – allosteric binding affinity, τ_B_ – intrinsic agonism, and αβ – allosteric cooperativity factor [36]

Following a HTS campaign of 81,600 compounds from the Vanderbilt Discovery Collection, a drug-like custom library, C1 (Fig. 1A) was identified as novel PAM at Y_4_R with a hit rate of PAMs of 0.015%. To consider the structural importance of the ethyl acetate moiety of the hit compound C1, a structural analog lacking this side chain was included in the study (C2, Fig. 1A). To monitor the effect of both compounds on G-protein activation in response to the endogenous peptide agonist PP, we first used Y_4_R-mediated Ca^2+^ flux assay in stably transfected COS-7_hY_4_R-eYFP_Δ6Gα_qi4−myr_ cells. Titration of the small molecules C1 and C2 induced a concentration-dependent potentiation of the native agonist PP response compared to DMSO vehicle, reaching a saturable effect at 3 µM for C1 and 30 µM for C2 (Fig. 1B). The functional data were analyzed with an operational model of allosterism by Aurelio et al. [36]. to quantitatively assess modulator affinity (K_B_), functional cooperativity (αβ), and allosteric agonism (τ_B_) between both compounds. C1 showed an affinity of 790 nM (pK_B_ ± SEM: 6.1 ± 0.18) to Y_4_R, whereas binding affinity of C2 was reduced by more than 10-fold compared to C1 with a K_B_ value of 8.5 µM (pK_B_ ± SEM: 5.1 ± 0.22). The positive cooperativity factor αβ indicates that the compounds enhance the ability of PP to promote Y_4_R-mediated G-protein activation, but with a higher effect observed for C1 (αβ = 4.8) than for C2 (αβ = 3.5). These data highlight the structural importance of the ethyl acetate moiety for PAM activity on the Y_4_R G-protein pathway, as both affinity and cooperativity were reduced for C2. The low τ_B_ values of 0.18 and 0.03 calculated for C1 and C2, respectively, indicate the absence of an intrinsic compound agonism (Fig. 1B) and confirm a pure PAM activity profile at Y_4_R (Fig. S1).

### Probe dependency and receptor selectivity of Y_4_R PAMs

**Fig. 2:**
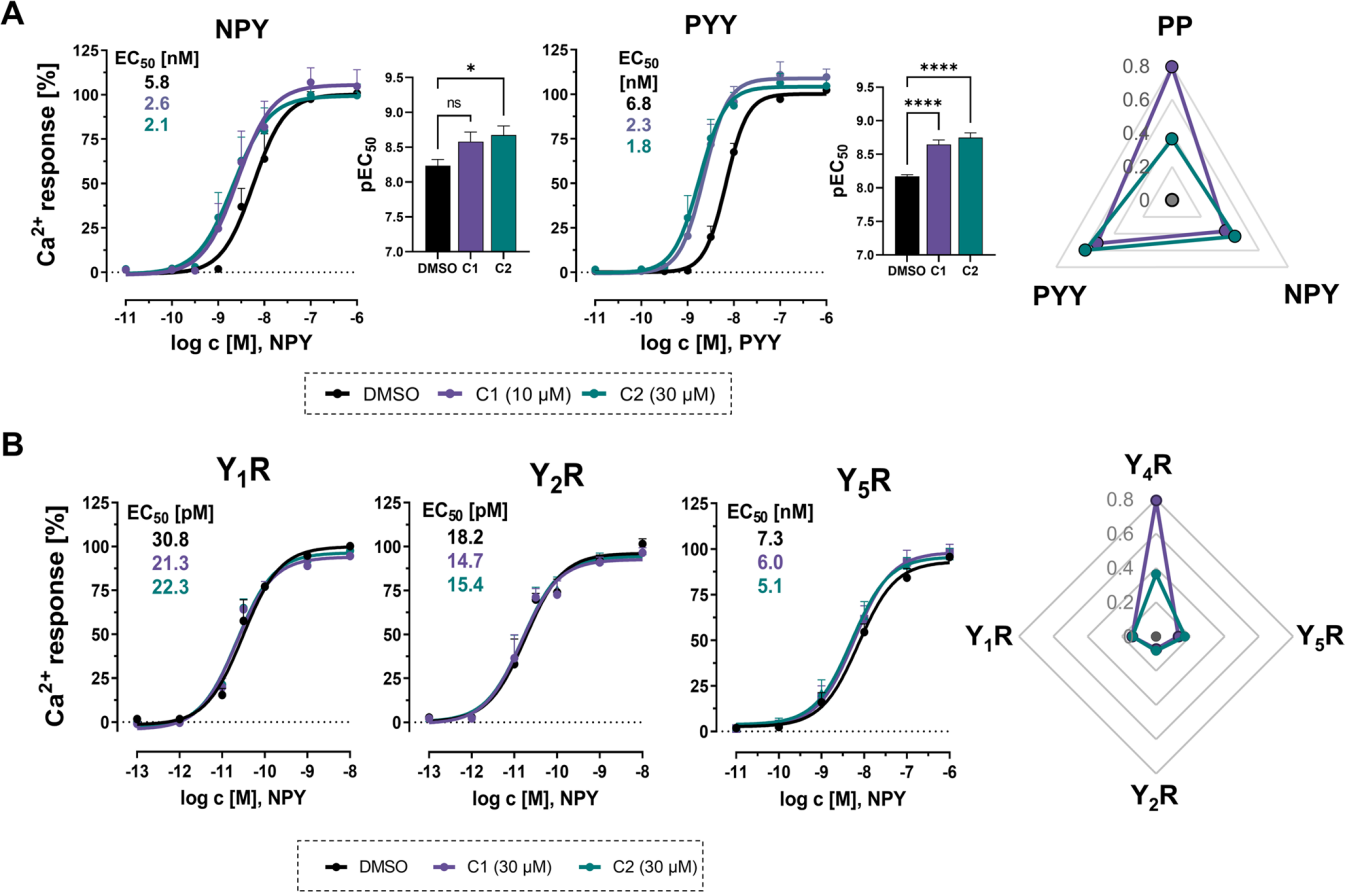
The PAMs C1 and C2 show a Y_4_R-selective potentiation of G-protein signaling independent of the peptide agonist. (**A**) Probe dependence effects of the compounds. Potentiation of Y_4_R G-protein signaling in the presence of 10 µM of C1 and 30 µM of C2 in response to the low-affinity ligands NPY and PYY. DMSO was used as control. Ca^2+^ flux assays were performed in COS-7_hY_4_R-eYFP_∆6Gα_qi4-myr_ cells. Statistical significance was determined by one-way ANOVA and Dunnett’s post test; ns, not significant; *, *P* < 0.05; ****, *P* < 0.0001. Radar plot illustration on ligand selectivity for the PAMs C1 and C2, shown as Δlog(E_max_/EC_50_) values based on data from Ca^2+^ flux experiments. (**B**) Effect of 30 µM of C1 or C2 on Y_1_R, Y_2_R, and Y_5_R G-protein activation. Y receptor selectivity was investigated in COS-7 cells stably co-expressing Y_1_R, Y_2_R, or Y_5_R, C-terminally fused to eYFP, and the chimeric G protein ∆6Gα_qi4-myr_. Radar plot presentation on Y receptor selectivity for the PAMs C1 and C2, shown as Δlog(E_max_/EC_50_) values based on NPY-activation data from Ca^2+^ flux experiments. Data are shown as mean ± SEM from *N* ≥ 3 independent experiments. Radar plots have been generated based on [48]

Next, receptor and ligand selectivity of the compounds was examined, as the Y_4_R belongs to a complex multiligand/multireceptor system comprising four GPCRs (Y_1_R, Y_2_R, Y_4_R, Y_5_R) and three peptide ligands (NPY, PYY, PP). To investigate NPY- or PYY-mediated Y_4_R activation, a saturating concentration of each compound (10 µM for C1 and 30 µM for C2) was used. Both compounds potentiate NPY- and PYY-mediated Y_4_R activity compared to the DMSO control, indicated by the leftward-shift of the curves in the presence of the compounds (Fig. 2A). Statistical evaluation revealed a significant effect for C2 on NPY-mediated Y_4_R signaling, while C1 exhibited a modest effect for NPY, notably weaker compared to the primary agonist PP. In contrast, PYY-induced G-protein activation at Y_4_R was significantly enhanced by C1 and C2 with EC_50_ shifts by about 3-fold. Next, the subtype selectivity of C1 and C2 among Y_1_R, Y_2_R, and Y_5_R was investigated by monitoring the receptor-mediated Ca^2+^ flux in COS-7 cells, stably expressing one specific receptor subtype. NPY was used as the endogenous, high-affinity ligand on these receptors. As indicated in Fig. 2B, C1 and C2 did not affect NPY-mediated G-protein signaling at Y_1_R, Y_2_R, and Y_5_R compared to the DMSO vehicle when used at a maximal concentration of 30 µM. This indicates a high functional selectivity of C1 and C2 for the Y_4_R subtype, as depicted in the radar plot for Y receptor selectivity (Fig. 2B), calculated as Δlog(E_max_/EC_50_) values [48].

In conclusion, we identified two PAMs that selectively potentiate Y_4_R G-protein signaling with the highest effect observed for the ligand PP. C1 showed a significantly enhanced PAM activity at Y_4_R compared to C2, highlighting the importance of its ethyl acetate moiety for PAM activity and affinity. Thus, only C1 was used for further characterization.

### C1 affects Y_4_R G-protein activity in G protein BRET and cAMP assays

**Fig. 3:**
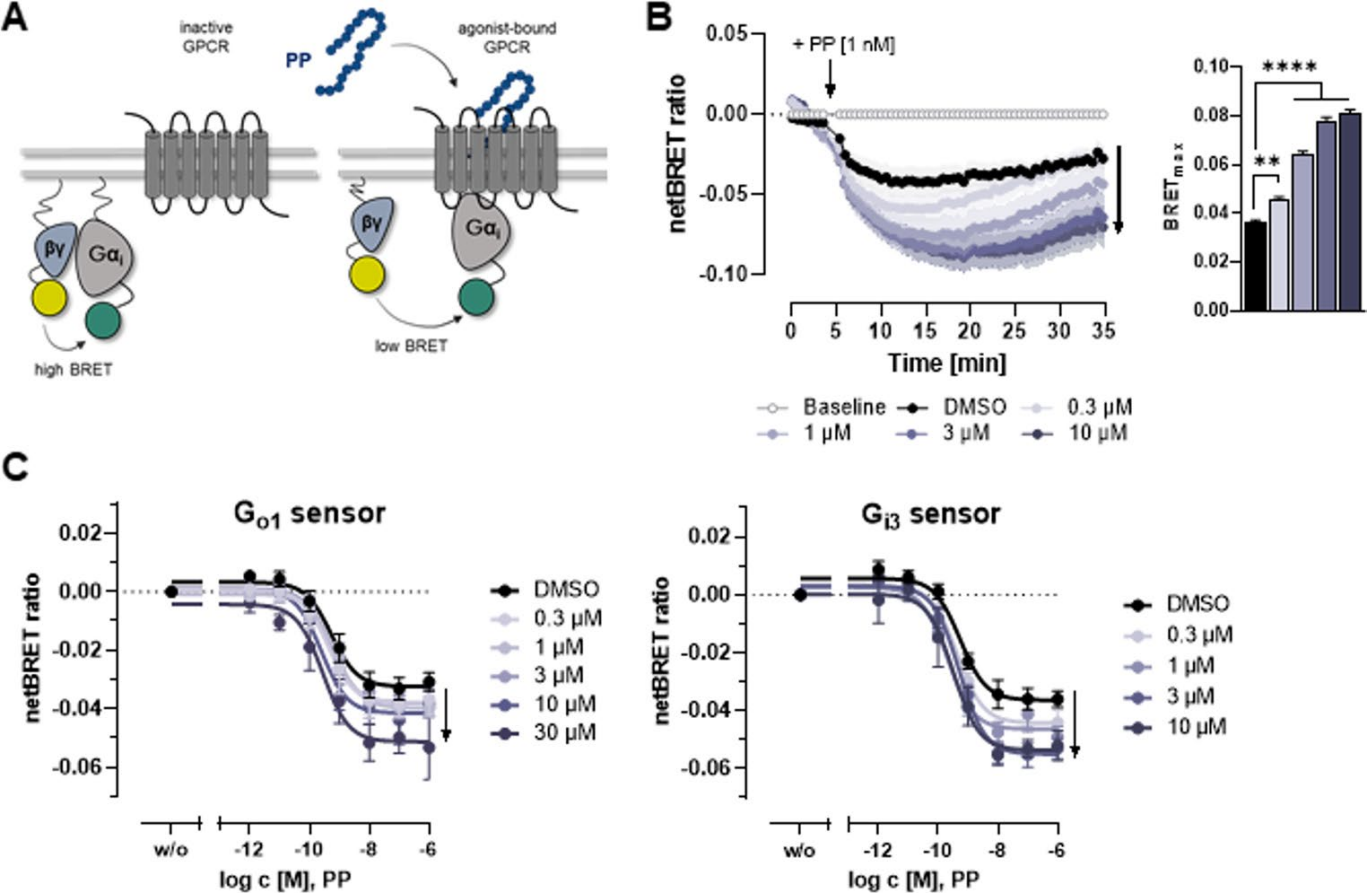
C1 potentiates native Y_4_R-mediated G_i/o_ signaling. (**A**) Assay principle of G protein BRET sensors according to Schihada et al. [37]. (**B**) Kinetic G protein BRET measurements using the G_i3_ BRET sensor. Y_4_R-mediated G-protein activity was detected in the presence of different concentrations of the PAM C1 and initiated by the addition of 1 nM of the agonist PP after about 5 min of baseline detection. BRET_max_ values are summarized as bar graphs. Statistical analysis was performed using one-way ANOVA with Dunnett’s post test, **, *P* < 0.01; ****, *P* < 0.0001. (**C**) Endpoint measurements of G-protein activity after 30 min of ligand stimulation using G_o1_ or G_i3_ BRET sensors in the presence of DMSO or different concentrations of C1. C1 increases the BRET_max_ of PP concentration-response curves compared to the DMSO control, indicated by black arrows. G protein BRET assays were conducted in COS-7 cells transiently co-transfected with Y_4_R and the respective G protein sensor. Data are shown as mean ± SEM from *N* ≥ 3 independent experiments

To further evaluate the effect of the PAM C1 at Y_4_R on its native G_i/o_ pathway and to exclude effects due to the chimeric G protein, two additional assays were used: G protein sensor BRET [37], which allows the direct measurement of G_i/o_ protein activity by monitoring the dissociation of the Gα and Gβγ moiety (Fig. 3A), and GloSensor cAMP assay [49], which uses a biosensor to monitor the intracellular second messenger cAMP in response to the activation of the Y_4_R-coupled G_i/o_ pathway. In kinetic G protein BRET assays using the G_i3_ sensor, C1 potentiated the PP-mediated Y_4_R G-protein activation as demonstrated by the reduction of the netBRET ratio in a concentration-dependent manner upon stimulation with a half-maximal concentration of PP (1 nM) over 30 min (Fig. 3B). Comparison of the BRET_max_ ratios indicate a significant increase in presence of the PAM, which is associated with an enhanced G-protein activity. In endpoint measurements, we have shown that C1 slightly, but not significantly, improves PP potency (leftward-shift of curves), but markedly increased the efficacy (BRET_max_) in G-protein dissociation for PP in a concentration-dependent manner compared to the DMSO control (Fig. 3C) using two different G protein sensors, G_i3_ and G_o1_ (Fig. S2). Thus, the data highlight an improved G-protein coupling in the presence of the PAM.

**Fig. 4:**
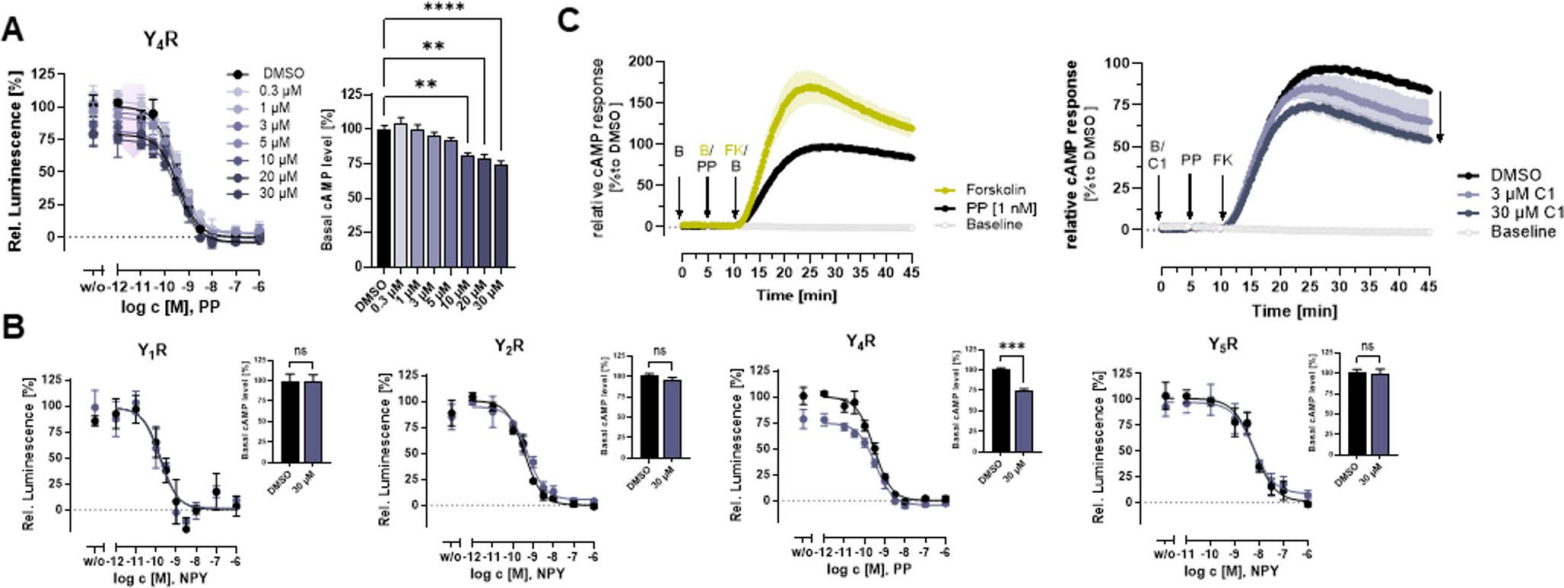
C1 selectively modulates basal Y_4_R activity. (**A**) Endpoint measurement of Y_4_R activity in cAMP GloSensor assay in COS-7 cells transiently expressing Y_4_R-eYFP and the GloSensor-22 F plasmid after 20 min of ligand stimulation in the presence of DMSO or different concentrations of C1. The basal cAMP levels relative to DMSO control was obtained from non-linear regression and are summarized as bar graphs. Statistical analysis was performed using one-way ANOVA with Dunnett’s post test, **, *P* < 0.01; ****, *P* < 0.0001. (**B**) Endpoint measurement of Y receptor activity in cAMP GloSensor assay in COS-7 cells transiently expressing Y_1_R, Y_2_R, Y_4_R, or Y_5_R and the GloSensor-22 F plasmid after 20 min of ligand stimulation in the presence of DMSO or 30 µM of C1. The basal cAMP levels relative to DMSO control was obtained from non-linear regression and are summarized as bar graph. Statistical analysis was performed using unpaired t-test, ns, not significant; ***, *P* < 0.001. (**C**) Time-dependent GloSensor cAMP assay in COS-7 cells transiently expressing Y_4_R-eYFP and the pGloSensor-22 F cAMP plasmid for investigating the native Y_4_R-mediated G_i/o_ protein signaling pathway. Cells were stimulated with DMSO or C1 subsequently before the measurement. The agonist PP or buffer (B, as control) were added after 5 min of baseline detection. Adenylyl cyclase activity was stimulated by the addition of 10 µM of forskolin (FK) after about 10 min. Luminescence was continuously monitored over 40 min and relative cAMP response was normalized to DMSO control. C1 reduces relative cAMP response compared to DMSO, indicated by the black arrow. Data are shown as mean from *N* ≥ 3 independent experiment; SEM is shown as shaded bands

To clarify how C1 acts further downstream on the native G_i_ signaling pathway, GloSensor cAMP assay has been used. For this purpose, the basal cAMP level of Y_4_R-expressing COS-7 cells was studied using the adenylyl cyclase activator forskolin. As shown in the concentration-response curve, increasing concentrations of PP lead to a reduction of Y_4_R-mediated cAMP levels (Fig. 4A, left). Notably, co-stimulation with increasing concentrations of C1 did not produce a leftward-shift of the concentration-response curve of PP at Y_4_R, but instead reduces the basal cAMP level (Fig. 4B), suggesting that C1 may also enhance the constitutive activity of Y_4_R [50]. In line with the Ca^2+^flux assay, this effect was not observed at Y_1_R, Y_2_R, or Y_5_R using a high concentration of C1, indicating a subtype-selective activity of C1 at Y_4_R (Fig. 4B). Additionally, we performed kinetic cAMP measurements at Y_4_R. Here, 10 µM of the adenylyl cyclase activator forskolin increases the intracellular cAMP levels in Y_4_R-expressing COS-7 cells. Stimulation with the peptide agonist PP (1 nM) in the presence of 0.3% DMSO reduced intracellular cAMP levels compared to the forskolin control, demonstrating G_i_ activation (Fig. 4C, left). To test the effect of the PAM C1 on a kinetic level, cells were stimulated with two concentrations of C1 (3 and 30 µM) or DMSO as control for 5 min. Afterwards, cells were stimulated with PP for another 5 min, before forskolin was added to increase intracellular cAMP levels. Compared to the DMSO control, C1 reduced the relative cAMP response, confirming the potentiation of PP-induced G_i_ signaling of Y_4_R (Fig. 4C, right). This effect saturated at low concentrations of C1, pointing towards a markedly higher inhibition of the adenylyl cyclase activity over time. These studies confirmed the PAM effect of C1 at Y_4_R on the native G_i_ pathway using G protein BRET as a direct read-out for G-protein activity, as well as in the second messenger cAMP assay.

### C1 slightly increases the affinity of the agonists PP and PYY to the Y_4_R orthosteric site

**Fig. 5:**
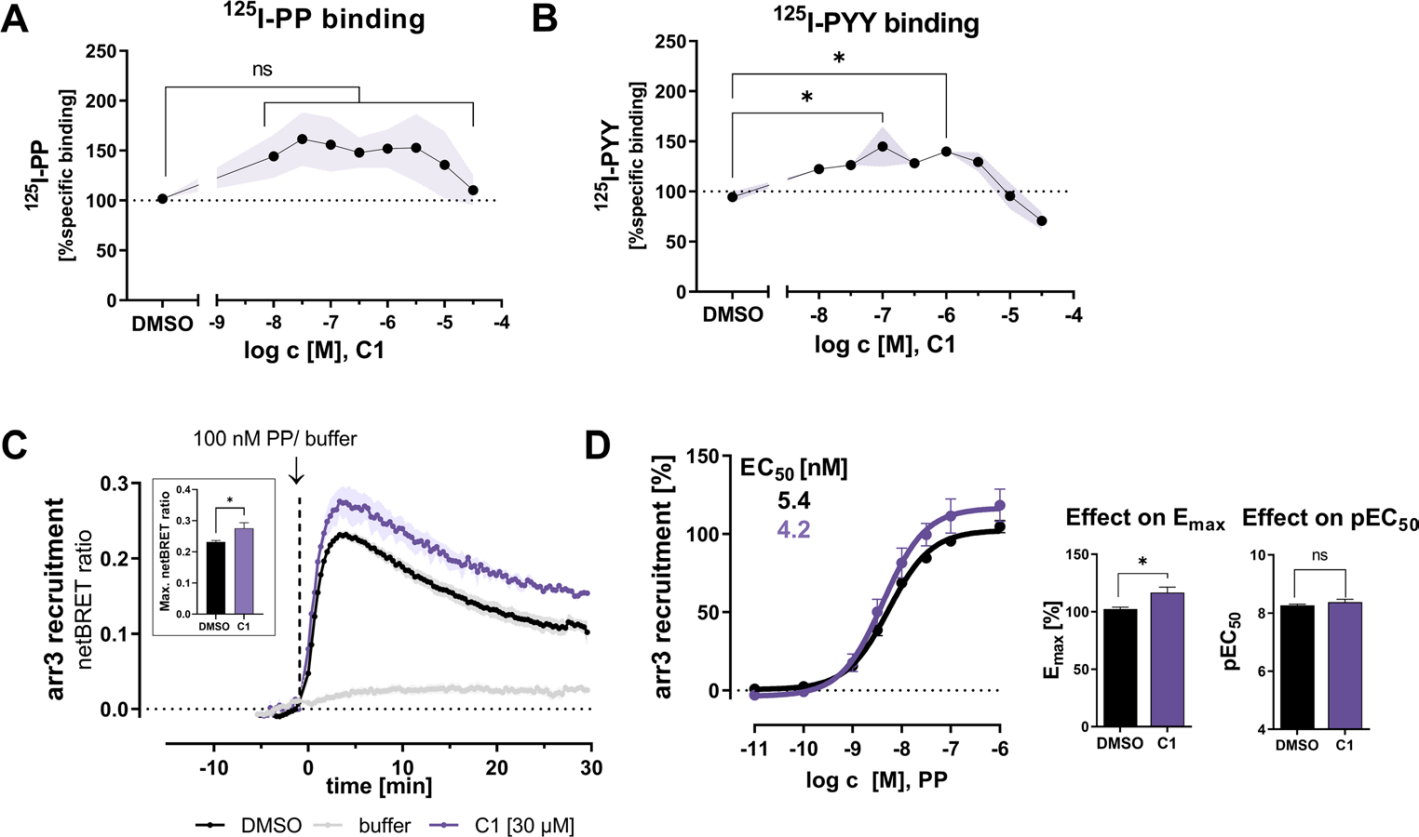
C1 slightly enhances ligand affinity and arrestin-3 recruitment to Y_4_R. (**A**,** B**) Effect of increasing concentrations of C1 on the binding of 30 pM ^125^I-PP (A) or 150 pM ^125^I-PYY (B) to membrane preparations from HEK293 cells stably expressing Y_4_R. Data represent the mean from *N* ≥ 3 independent experiments, SEM are shown as shaded bands. Statistical significance was determined by one-way ANOVA with Dunnett‘s post test; ns, not significant; *, *P* < 0.05. (**C**,** D**) BRET analysis was used to study the effect of 30 µM of C1 on arrestin-3 recruitment to Y_4_R using COS-7 cells, transiently expressing Y_4_R-Rluc8 and Venus-arrestin-3. Arrestin-3 recruitment kinetics were monitored over 30 min after stimulation of the receptor with 100 nM of the agonist PP. Data represent the mean from *N* ≥ 2 independent experiments, SEM are shown as shaded bands (C). Effect of 30 µM of C1 on PP concentration-response curves was measured after 5 min of agonist stimulation. DMSO is used as control. Data are shown as mean ± SEM from *N* ≥ 3 independent experiments (D). Statistical evaluation was performed using unpaired student’s t-test, ns, not significant; *, *P* < 0.05

The presence of allosteric modulators has been shown to affect the conformational landscape of its cognate receptor, which can modulate the affinity and binding mode of the orthosteric agonist. Therefore, the effect of C1 on the affinity of the peptide ligands PP and PYY to Y_4_R membrane preparations was investigated by radioligand binding assay using ^125^I-PP and ^125^I-PYY. Y_4_R membranes were incubated with C1 and the radioligand for about 4 h until equilibrium was reached. C1 slightly enhanced the binding of ^125^I-PP to Y_4_R membranes for concentrations up to 10 µM compared to the DMSO control, while this effect was not observed at the highest concentration of 30 µM (Fig. 5A). A similar effect of C1 was observed for the binding of ^125^I-PYY to Y_4_R membranes at equilibrium (Fig. 5B). Here, C1 increased the affinity of ^125^I-PYY to Y_4_R up to a concentration of 3 µM, whereas binding of PYY was unaffected at high compound concentrations (10 µM and 30 µM).

### Effect of C1 on arrestin-3 recruitment to Y_4_R

The Y_4_R was previously observed to recruit arrestin-3 after agonist stimulation [51]. Therefore, we assessed the effect of C1 on the arrestin-3 recruitment pathway in addition to G-protein signaling. The Y_4_R-arrestin-3 interaction was studied by BRET assay in COS-7 cells transiently expressing Y_4_R-*Renilla* luciferase 8 (Rluc8) and Venus-tagged arrestin-3 using two different set-ups: kinetic measurements, at which PP-induced arrestin-3 recruitment was constantly detected over 30 min, and endpoint measurements, at which arrestin-3 recruitment was detected 5 min after stimulation with increasing PP concentrations. In kinetic experiments, treatment of COS-7 cells with 30 µM of C1 induced a significant increase in the maximal netBRET ratio of arrestin-3 recruitment to Y_4_R compared to DMSO after stimulation with 100 nM of the agonist PP. Arrestin-3 was mainly recruited to Y_4_R within the first 10 min after PP stimulation and started to dissociate from the receptor shortly afterwards (Fig. 5C). Therefore, BRET signals from concentration-dependent measurements were detected after 5 min of agonist stimulation. Compared to DMSO, we observed a significant enhancement of the efficacy (E_max_) in arrestin-3 recruitment in the presence of high concentrations of C1 (30 µM) by about 20%, while potency of arrestin to Y_4_R remained unaffected in the presence of C1 with an EC_50_ value of 4.2 nM (pEC_50_ ± SEM: 8.38 ± 0.09) (Fig. 5D). This suggests a positive allosteric effect of C1 in the arrestin-3 recruitment pathway.

### Identification of a potential binding mode of C1 at Y_4_R obtained by mutagenesis and computational docking

To better understand the effect of C1 on PP modulation, we characterized the potential binding pocket of C1 to Y_4_R by mutagenesis and computational docking. To get a first hint on Y_4_R segments that are important for the PAM effect of C1, different Y_4_R/Y_1_R chimeric receptors were used. The PAM effect was studied as the relative potentiation of a low-efficacy PP concentration (PP EC_30_) at each chimera in a Ca^2+^ flux-based assay set-up using COS-7 cells transiently expressing a chimeric receptor variant and the G protein chimera ∆6Gα_qi4−myr_.

#### Y_4_R/Y_1_R chimeras highlight the relevance of residues within transmembrane (TM) helix 1, TM2, extracellular loop 1 (ECL1), TM6, and TM7 of Y_4_R for C1 activity

**Fig. 6:**
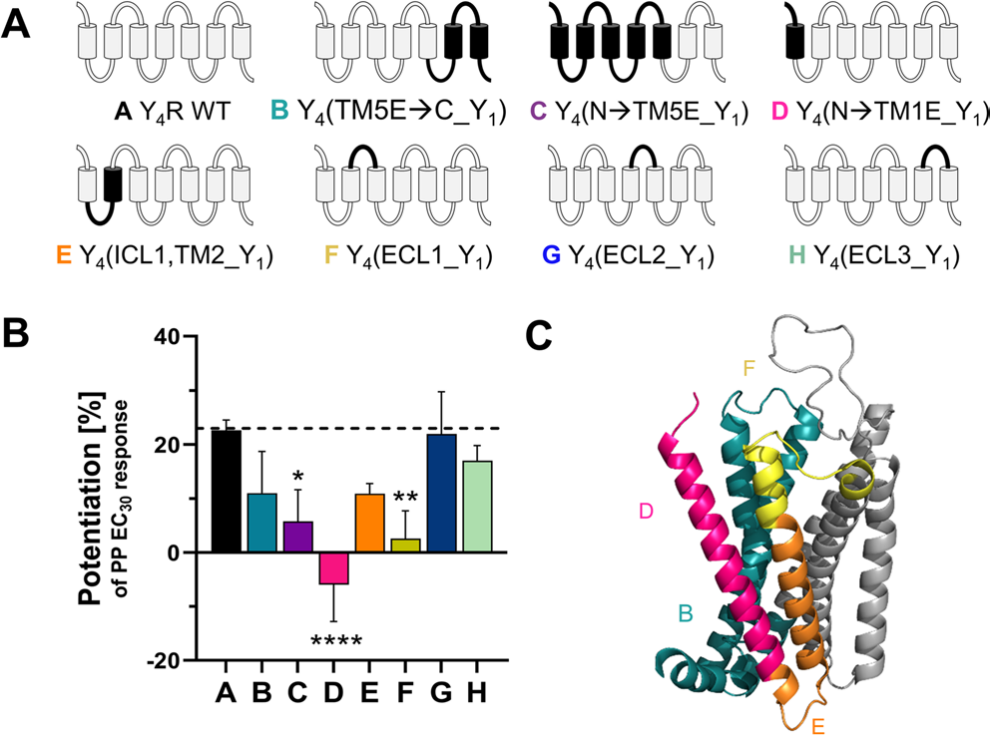
Important Y_4_R segments for C1 activity identified using Y_4_R/Y_1_R chimeras. (**A**) Schematic representation of Y_4_R/Y_1_R chimeras. Grey color represents Y_4_R segments, Y_1_R segments are colored in black. (**B**) Relative potentiation of the PP EC_30_ response in the presence of 10 µM of C1 compared to DMSO. Mutagenesis studies were performed using Ca^2+^ flux assay in transiently transfected COS-7 cells expressing one Y_4_R/Y_1_R chimera and the chimeric G protein ∆6Gα_qi4-myr_. Data are shown as mean ± SEM from *N* ≥ 3 independent experiments. Statistical significance was determined by one-way ANOVA with Dunnett’s post test; *, *P* < 0.05; **, *P* < 0.01; ****, *P* < 0.0001. (**C**) Highlighting of important Y_4_R segments according to the mutagenesis studies using Y_4_R/Y_1_R chimeras (PDB: 7X9C)

The use of Y_4_R/Y_1_R chimeras allows the identification of relevant receptor segments and amino acid residues differing between the Y_1_R and Y_4_R subtype. Here, we investigated the effect of C1 (10 and 30 µM) on the potentiation of the PP EC_30_ response at seven different Y_4_R/Y_1_R chimeras (Fig. 6A, Table S1). The absolute potentiation of PP response induced by 10 µM of C1 versus DMSO is illustrated as bar graphs (Fig. 6B). At Y_4_R WT, C1 induced a relative potentiation of the PP EC_30_ response by 23% in transiently transfected COS-7 cells. The C-terminal exchange of Y_4_R segments, ranging from intracellular loop 3 (ICL3) to the C terminus, indicates the involvement of some residues in the Y_4_R regions TM6-ECL3-TM7 for C1 activity, as the PAM effect is slightly reduced at this chimera (chimera B; Fig. 6). The exchange of a large Y_4_R segment, covering the N terminus up to the end of TM5 by the corresponding Y_1_R sequence, resulted in a significant loss of PAM activity, and highlights the importance of the N-terminal region for the PAM activity of C1 (chimera C; Fig. 6). Next, Y_4_R/Y_1_R chimeras with smaller segmental exchanges at the N-terminal receptor part were studied (chimera D and E; Fig. 6). At chimera D, in which the N terminus and TM1 of Y_4_R are replaced by the corresponding sequence of Y_1_R, a complete loss of PAM activity was found for C1. Thus, TM1 seems to be strongly involved in the interaction with the compound. The replacement of ICL1 and TM2 of Y_4_R resulted in a moderate loss of PAM activity with a remaining potentiation of about 11% (chimera E; Fig. 6). Next, Y_4_R loop chimeras were investigated. Chimera F contains a replacement of the Y_4_R ECL1 by the loop of the Y_1_R. This exchange led to a significant loss of C1 activity by about 10-fold, indicating the relevance of residues within ECL1 for C1 activity. The exchange of the large ECL2 of Y_4_R by the corresponding loop of the Y_1_R revealed a relative potentiation of 22% comparable to the WT and is negligible for compound activity (chimera G; Fig. 6). The replacement of the ECL3 of Y_4_R by the ECL3 of Y_1_R did not exhibit a clear loss of C1 PAM activity with a potentiation of PP EC_30_ response to 17% (chimera H; Fig. 6). Thus, these data indicate that TM1, TM2, ECL1, TM6, and TM7 might contain relevant residues required for C1 PAM activity (Fig. 6C).

### Single-point mutagenesis in combination with computational docking revealed potential binding poses of C1 at Y_4_R

#### Single point mutagenesis identified important Y_4_R residues for PAM activity

**Fig. 7:**
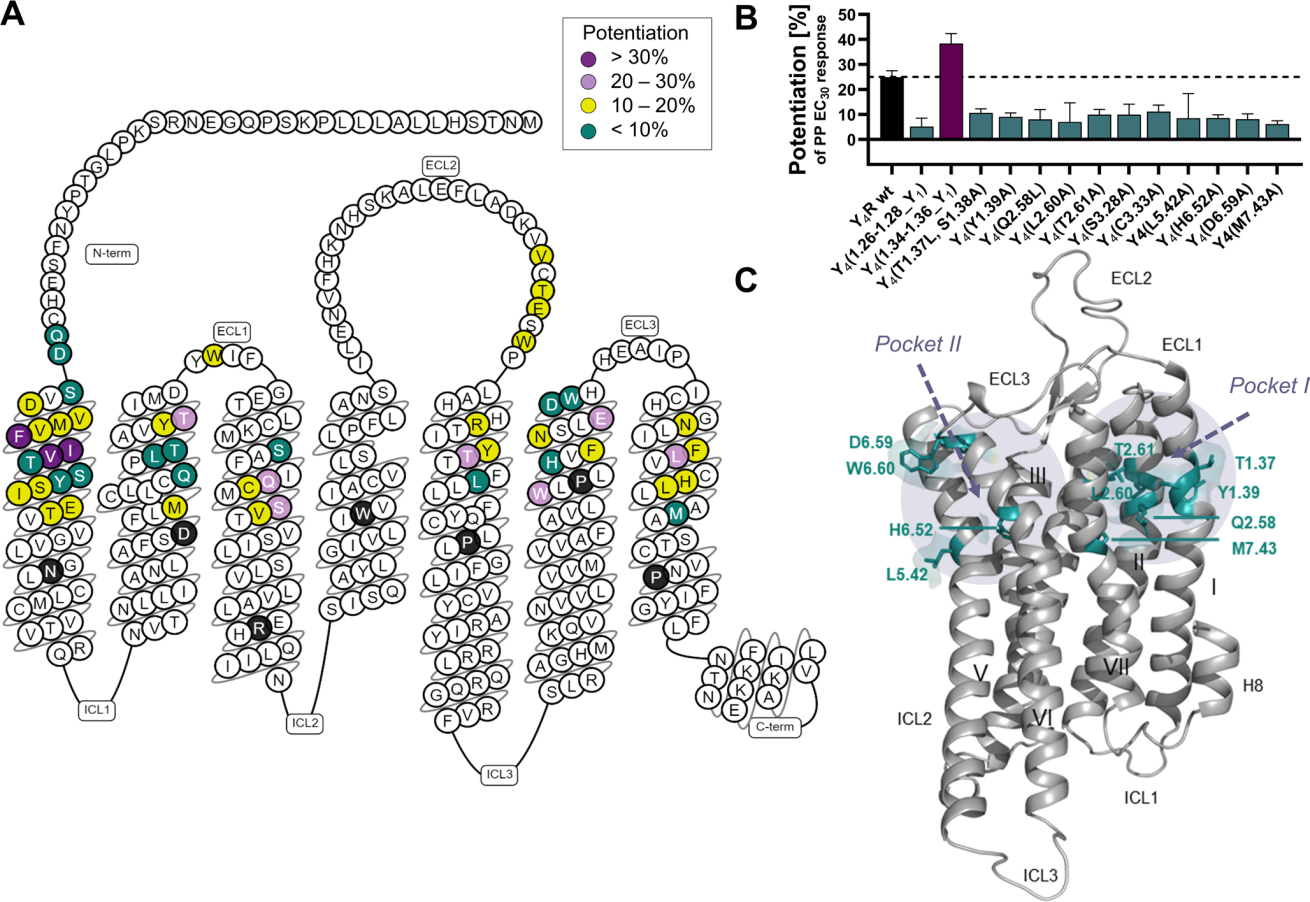
Y_4_R residues important for C1 activity investigated by mutagenesis. (**A**) Snake plot of Y_4_R highlighting the tested residues for C1 PAM activity (adapted from GPCRdb.org). The color code represents the potentiation of the PP EC_30_ response at Y_4_R variants in the presence of 30 μM of C1 investigated by Ca^2+^ flux assay: potentiation > 30% (dark purple), 20 – 30% (lilac), 10 – 20% (yellow), < 10% (cyan). Residues shown in black represent the most conserved residue per helix (x.50) according to the Ballesteros-Weinstein nomenclature. [52] (**B**) Relative potentiation of the PP EC_30_ response in the presence of 30 μM of C1 at the most important Y_4_R variants. Mutagenesis studies were performed by using Ca^2+^ flux assay in transiently transfected COS-7 cells expressing a Y_4_R variant and the chimeric G protein Δ6Gα_qi4-myr_. Data are shown as mean ± SEM from N ≥ 3 independent experiments. (**C**) Structural model of Y_4_R (PDB: 7X9C) highlighting residues important for C1 PAM activity. Y_4_R is shown in cartoon representation (grey), relevant residues with a loss of PAM activity are presented as cyan sticks. Two potential binding pockets according to mutagenesis are highlighted in light lilac,* Pocket I* and *Pocket II*

To identify specific Y_4_R positions involved in the interaction with C1, a mutagenesis scan of Y_4_R positions located at the extracellular interface was performed. Here, the effect of 10 µM and 30 µM of C1 on the potentiation of the PP EC_30_ response at 41 Y_4_R variants was investigated by Ca^2+^ flux assay (Table S1). For the screen, Y_4_R residues that are known to be important for the endogenous peptide ligand PP or a reported Y_4_R allosteric antagonist have been included [7, 35]. At Y_4_R WT, C1 induced a relative potentiation of the PP EC_30_ response by about 25% in the presence of 10 µM and 30 µM of C1 (Table S1). As shown for Y_4_R/Y_1_R chimeric receptors, TM1, TM2 and ECL1 have been shown to be important receptor domains for C1 activity. A deeper insight into these regions revealed that Ala mutations of Y1.39, Q2.58, L2.60, and T2.61, as well replacements of the regions 1.26–1.28 and 1.37–1.38 by Y_1_R residues significantly reduced the PAM activity of C1, while mutations of the residues 1.30–1.33, 1.40–1.43, M2.53, Y2.64 and W2.70 only resulted in a moderate loss of C1 activity. (Table S1, Fig. 7A). Mutation of residues 1.34–1.36 by the corresponding residues of Y_1_R (Thr-Leu-Ala) induced an increase in C1 activity with a potentiation of PP EC_30_ response by 38% (Table S1, Fig. 7A). In TM3, mutation of S3.28 and C3.33 affected C1 activity with less than 10% PAM activity, while the variants S3.35 A and V3.36 A only moderately affect C1 activity. In TM5, residue L5.42 exhibited a clear loss in C1 activity if mutated to Ala (Table S1, Fig. 7). As indicated in Fig. 6, TM6 and TM7 are important receptor domains for C1 activity. Due to the sequence similarity of Y_4_R and Y_1_R within TM6 and TM7 (Fig. S3), identical residues between both subtypes were tested individually by single amino acid mutagenesis. Here, the Y_4_R residues H6.52, D6.59, W6.60, and M7.43 were identified as important amino acids for C1 activity if mutated to Ala with less than 10% remaining PAM activity (Table S1, Fig. 7A, B). Y_4_R residues without a clear effect on C1 potentiation are summarized in Table S1. Y_4_R residues important for C1 PAM activity according to mutagenesis are visualized in Fig. 7C and reveal two potential binding pockets of C1. *Pocket I* is located within the Y_4_R TM helical bundle and formed by residues within TM1, TM2, and TM7. The second cluster (*Pocket II)* is located between TM3, TM5 and TM6, however, in this cluster most residues are pointing outside the binding pocket, making this pocket improbable.

#### Computational docking verifies binding to an Y_4_R allosteric pocket

**Fig. 8:**
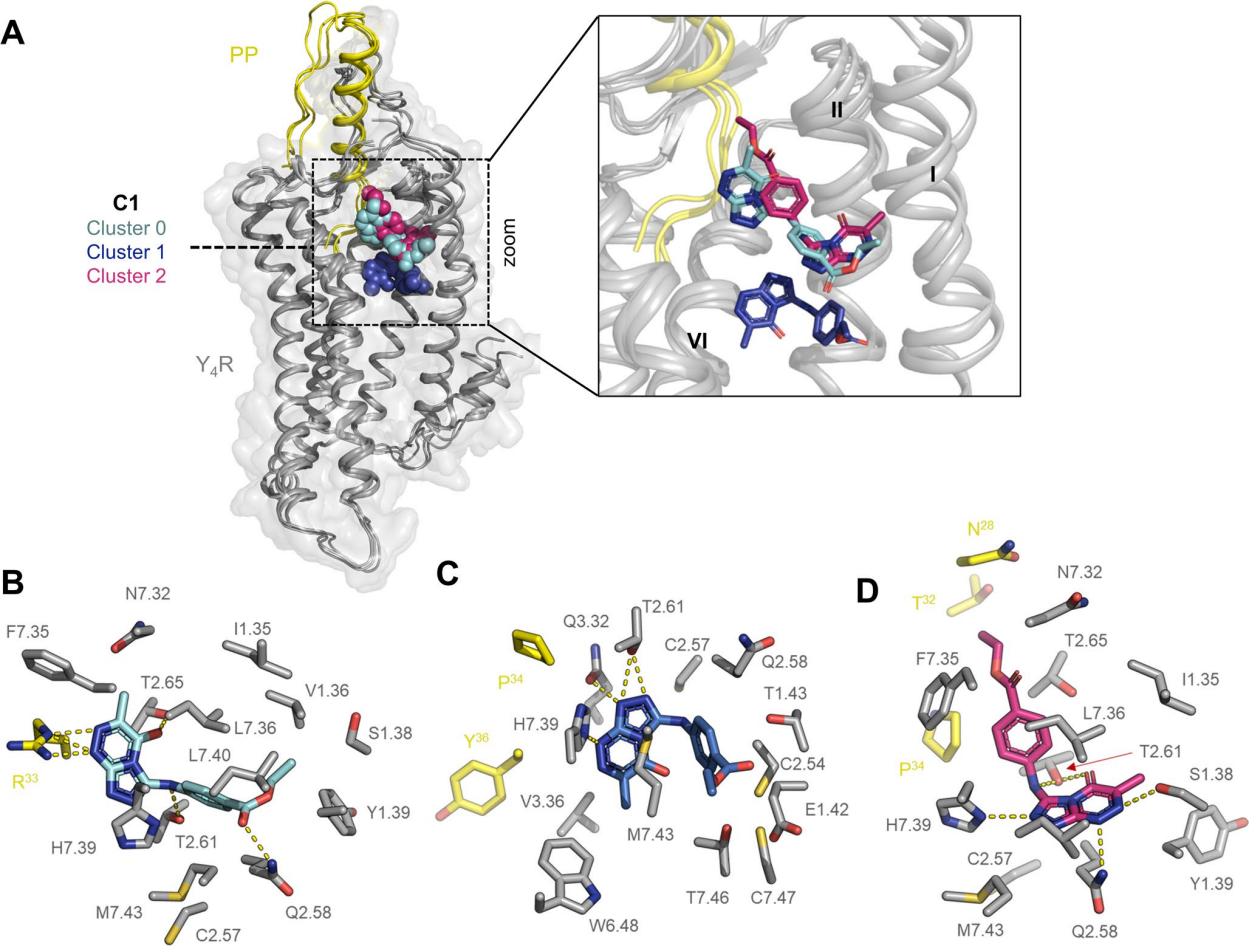
Proposed binding poses of C1 to Y_4_R in three clusters within the 7TM bundle. (**A**) Overlay of C1 binding clusters at Y_4_R. Y_4_R and PP are shown in cartoon representation in grey and yellow, respectively. TM7 is omitted for better illustration. C1 is shown as cyan (cluster 0), blue (cluster 1), or pink (cluster 2) spheres/sticks. For Rosetta docking, the PP-Y_4_R-G_i1_ cryo-EM structure (PDB: 7X9C) was used as template for an optimized PP-Y_4_R structure [7]. (**B-D**) Polar environment of C1 in cluster 0 (B), cluster 1 (C), and cluster 2 (D). Dashed yellow lines represent polar interactions between Y_4_R residues (grey sticks) and C1 (cyan, blue, pink sticks). PP residues in the polar surrounding of C1 are shown as yellow sticks. Receptor residues are designated according to the nomenclature of Ballesteros-Weinstein [52]

To obtain a more precise understanding on the binding mode of C1 at Y_4_R, computationally induced-fit docking of the compound into an optimized Y_4_R-PP cryo-EM structure (PDB: 7X9C) [7] was performed based on the mutagenesis data. Here, three potential binding clusters of the compound at Y_4_R have been obtained. In all clusters, the PAM is placed in the core of the Y_4_R 7-transmembrane (7TM) bundle near the C-terminal binding interface of PP, in a pocket mainly formed by residues in TM1, TM2, and TM7 (Fig. 8A). Thus, the molecular docking supports a binding of C1 in *Pocket I* (Fig. 7C), as suggested by mutagenesis. In cluster 0 and cluster 2, C1 occupies a similar allosteric pocket, while the compound in cluster 1 is located deeper within the helical bundle (Fig. 8A). Fig. 7B – D show the polar receptor and ligand environment of the compound in the three different clusters.

In cluster 0, the ethyl benzoate moiety of the compound is located in a crevice formed by TM1, TM2, and TM7. The ethyl acetate side chain of C1 points towards TM1 and TM2 of Y_4_R and is engaged in a polar interaction with Q2.58 of Y_4_R. Therefore, removal of the ethyl acetate side chain in C2 might destabilize the binding by the loss of a strong polar contact, which is indicated by a reduced affinity of C2 compared to C1. The triazine derivatives of C1 are orientated between TM2 and TM7 near the C terminus of the endogenous peptide ligand PP. Here, the aromatic heterocycle backbone is involved in polar interactions with R^33^ of PP and the Y_4_R residues T2.65 and T2.61 in TM2 (Fig. 8B). Based on the mutagenesis data, mutation of the residues T2.61 and Q2.58 resulted in a severe loss of PAM activity, while the variant T2.65A shows no effect on the PAM effect, indicating no relevance of this residue for C1 activity (Fig. 7, Table S1).

In cluster 1, C1 forms a U-shaped conformation and is located deeper within the 7TM helical bundle compared to cluster 0 and 2. Here, the ethyl acetate side chain is oriented between TM1 and TM7 in a polar pocket formed by residues T1.43, C2.54, C7.47, and T7.46. The aromatic heterocycles are located deep inside the bundle in a cavity shaped by residues within TM2, TM3, and TM7 (Fig. 8A, C). Here, the nitrogen atoms of the triazole form direct polar interactions with the Y_4_R residues T2.61, Q3.32, and H7.39. While mutation of position T2.61 to Ala markedly reduced the potentiation of PP-mediated Y_4_R G-protein signaling, mutation of the polar residue Q3.32 had no effect on C1 activity (Fig. 7A, B). In cluster 1, C1 is in close proximity to the very C terminus of PP (Y^36^-amide) highlighting the deep binding mode of the compound in this cluster (Fig. 8C).

In cluster 2, C1 binds within the 7TM helix bundle and is located in a similar pocket than the compound in cluster 0, but with a reversal orientation. Here, the ethyl benzoate group of C1 points up towards the extracellular receptor region and is located parallel to the C terminus of the endogenous agonist PP, which might allow the compound to form potential backbone interactions with the peptide agonist PP (T^32^/N^28^). Removal of the ethyl acetate as in C2 could therefore destabilize the PP-bound conformation and reduce the allosteric cooperativity with PP, which might explain the reduced PAM activity of C2 compared to C1 at Y_4_R (Fig. 1). Additionally, C1 might form weak hydrophobic interactions with P^34^ of PP, further stabilizing the active conformation. In this cluster, the heterocycles of C1 are positioned between TM1, TM2, and TM7 and are engaged in a polar interaction network with residues S1.38, T2.61, and Q2.58 (Fig. 8D). Mutation of positions T2.61 and Q2.58 to Ala or Leu, respectively, resulted in a remarkable loss of PAM activity, indicating the importance of the polar residues for C1 activity. Position S1.38 showed a reduction of PAM activity by more than 60% in the double-mutated variant Y_4_(T1.37L, S1.38A), highlighting the significance of the polar Y_4_R residues for binding of C1 in cluster 2. Similar as in cluster 1, residue H7.39 forms a polar interaction with the nitrogen of the triazol ring of C1. With regard to the mutagenesis data, this position seems to be of minor relevance for C1 with about 70% remaining PAM activity of the Ala variant compared to the WT, while a mutation to Pro reduced the PAM activity by more than 50% (Fig. 7A, Table S1). This might indicate that the introduction of Pro might change the orientation of the helix by inducing a kink, and thus hampers important nearby interactions.

Based on these data, binding of C1 to Y_4_R in cluster 2 is most likely as it fits well with mutagenesis data and shows the highest mutagenesis agreement score based on interface energy calculations (Table S2), while binding in cluster 1 is improbable.

#### Loss- and *gain-of-function* variants support the binding mode of C1 to Y_4_R in cluster 2

**Fig. 9:**
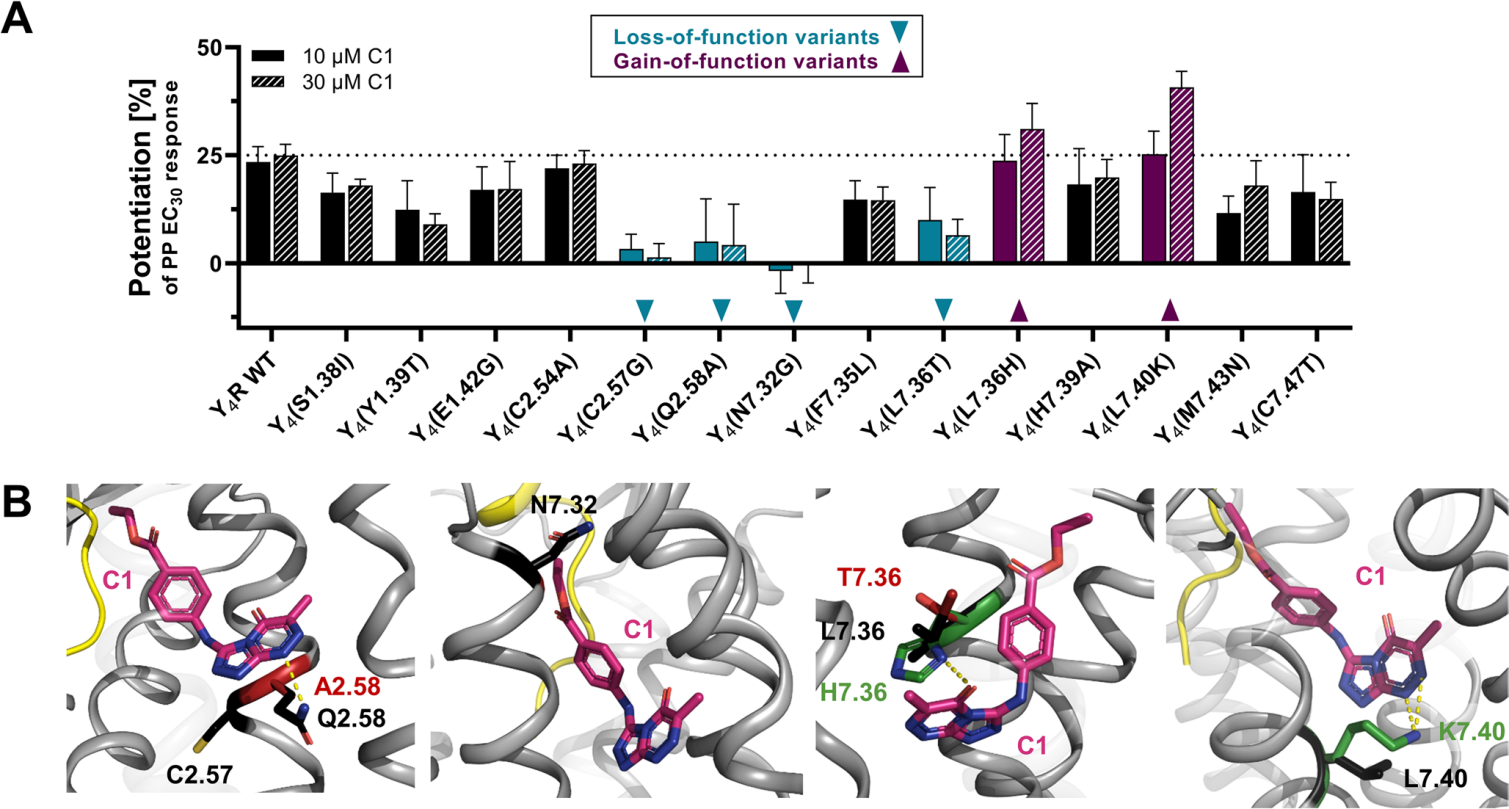
PAM effect of C1 at new Y_4_R variants. (**A**) Potentiation of the PP EC_30_ response at novel Y_4_R variants in the presence of 10 and 30 µM of C1 investigated by Ca^2+^ flux assay in COS-7 cells transiently expressing Y_4_R or variants and the chimeric G protein ∆6Gα_qi4-myr_. Data are shown as mean ± SEM from *N* ≥ 3 independent experiments. Variants, at which the PAM effect of C1 is lost, are highlighted in cyan and designated as *loss-of-function* variants, while variants with improved potentiation of the PP response are referred to as *gain-of-function* variants and are colored in purple. (**B**) Verification of *loss-of-function* and *gain-of-function* variants for the PAM C1 in the most likely binding cluster of C1 (cluster 2). The compound is shown in pink stick representation, Y_4_R is shown as grey cartoon. Specific Y_4_R residues are shown as sticks: black – native residue, green – *gain-of-function* variant, red – *loss-of-function* variant. Polar interactions are shown as dashed yellow lines

To further verify the most likely binding mode of C1 to Y_4_R, Y_4_R residues that are in close proximity to the compound and might positively affect C1 activity have been predicted. To functionally evaluate the predicted variants, 14 Y_4_R mutants have been created using single-point mutagenesis PCR and tested for G-protein signaling and membrane expression (Fig. S4). To obtain insight into the functional relevance of the receptor variants for C1 activity, potentiation of the PP EC_30_ response was studied in presence of 10 and 30 µM of C1. Here, the Y_4_R variants S1.38I, E1.42G, C2.5A, H7.39A, M7.43N, and C7.47T have a negligible effect on C1 activity, while Y1.39T and F7.35 L reduced the PAM activity of C1 by about 50% (Fig. 9A). For two variants L7.36H and L7.40K, the PAM activity is increased at a maximal concentration of 30 µM, identifying them as weak *gain-of-function* variants. Interestingly, we also identified four variants (C2.57G, Q2.58A, N7.32G, L7.36T) that markedly reduced C1 PAM activity (Fig. 9A).

In cluster 0 and 2, C1 directly interacts with the Y_4_R residue Q2.58, while C1 in cluster 1 is not involved in interactions with Q2.58 (Fig. 8B – D). Mutation to Ala in cluster 0 and 2 might disturb the polar interaction and consequently weakens the Y_4_R-PAM interaction (Fig. 9B). Additionally, mutation of the Cys residue at position 2.57 to Gly hampers the activity of C1 at Y_4_R in activity studies (Fig. 9A). Gly residues in the 7TM domain are known as ‘helix breaker’ [53, 54]. Thus, this mutation might destabilize the helical structure and might interrupt nearby interactions of C1 with Y_4_R (e.g. polar interaction with Q2.58 in cluster 2), which can strongly reduce the activity of the compound (Fig. 9A). Another *loss-of-function* Y_4_R variant is N7.32G. Here, the Gly residue might destabilize the C1-PP-Y_4_R interaction, indicating that the polar Asn residue is relevant at this position, even if it is not involved in a direct interaction with C1. The Y_4_R variants L7.36H and L7.40K were predicted as *gain-of-function* variants for C1 activity. We have shown that a concentration of 10 µM of C1 potentiated PP-mediated Y_4_R response by 25% at H7.36 comparable to the WT receptor, whereas 30 µM of C1 increased the PAM activity to 30%. In contrast, a Thr mutant at position 7.36 reduced the PAM activity of C1 (Fig. 9A), highlighting the importance of this position for the compound. As shown in Fig. 9B, His at position 7.36 might form an additional polar bond with C1 in cluster 2 and shows significant van der Waals overlaps, thus tightening the binding of the compound to its Y_4_R allosteric pocket. In cluster 1, the compound binds too deep within the 7TM bundle to contact position 7.36. In cluster 0, a mutation of L7.36 to His forms van der Waals interactions with C1, but is not able to make an additional polar interaction (data not shown), strengthening the binding mode of C1 as shown in cluster 2. Another relevant position is L7.40. A Lys at position 7.40 markedly enhanced the activity of the PAM compared to the native residue Leu (Fig. 9A). In cluster 2, mutation to Lys at position 7.40 of Y_4_R might form an additional polar interaction with C1, thus stabilizing the active Y_4_R conformation. In conclusion, we identified several *loss*- and *gain-of-function* variants that are unique to cluster 2, supporting cluster 2 as the most likely binding mode of C1 to Y_4_R.

## Discussion

The Y_4_R, a peptide GPCR, is involved in the regulation of critical physiological processes, including energy homeostasis, food intake, colonic transit, and gut motor function [29, 55, 56]. GPCR activation can be modulated by different types of ligands acting through orthosteric or allosteric receptor sites. Notably, Y_4_R agonists or PAMs have emerged as potential anti-obesity drugs [20, 57–59]. Within the last decades, many efforts have been made to identify ligands that are selective for the Y_4_R subtype, and thus forward the Y_4_R as a clinically relevant drug target for metabolic disorders. Obinepitide (TM-30339, 7TM Pharma) [60], a synthetic chimeric variant of the peptides PP and PYY, acts as a dual Y_2_R/Y_4_R agonist and has undergone phase II clinical trials for obesity treatment. However, its peptide nature poses challenges regarding its metabolic stability and oral bioavailability in vivo. To address these issues, efforts have been made to develop stabilized PP analogs through PEGylation [61, 62] or lipidation [62–64]. Additionally, several Y_4_R agonists derived from the C-terminal pentapeptide of the NPY peptides have been described. The first peptides derived by this approach, e.g. GR231118, showed poor selectivity within the Y receptor family [65]. However, recent studies report on C-terminally-derived pentapeptides with high and selective Y_4_R activity [24, 66–68]. Another promising approach is the use of Y_4_R positive allosteric modulators, which precisely modulate agonist-induced Y_4_R signaling through an allosteric pocket. Low-molecular weight allosteric compounds offer improved stability and bioavailability compared to unmodified peptides with reduced side effects as they enhance the effects to endogenous ligands, while maintaining native spatiotemporal signaling patterns [69, 70]. We have already reported on the identification of several Y_4_R allosteric modulators. The first Y_4_R PAM was niclosamide, which showed a poor Y receptor selectivity and was further reported to act as allosteric antagonist at the metabotropic glutamate 1 receptor (mGluR1) [28, 71]. Advanced Y_4_R-selective allosteric modulators include the agonistic PAM (ago-PAM) tBPC and the allosteric antagonist *(S)*-VU0637120 [27, 35]. VU0506013 has been reported as Y_4_R pure PAM that modulates the G-protein pathway without affecting ligand binding [39]. In this study, we identified C1 (VU0610218) as a structurally novel Y_4_R PAM with nanomolar binding affinity and a high allosteric cooperativity. Compared to VU0506013, which is characterized by a higher binding affinity (K_B_) to Y_4_R, C1 shows a markedly improved positive allosteric cooperativity with the endogenous agonist PP. Thus, both Y_4_R PAMs have different characteristics with respect to their allosteric behavior. In addition to the G-protein pathway (Figs. 1 and 3), C1 was shown to increase the efficacy of arrestin-3 recruitment to Y_4_R (Fig. 5). Consequently, C1 does not exhibit pathway selectivity towards the two major pathways for GPCRs [1] and acts as potentiator at the G-protein signaling and arrestin-3 recruitment pathway. In the G-protein pathway, C1 affects the potency and efficacy of the agonist PP, while in the arrestin recruitment pathway, C1 shows an efficacy-driven effect. The increased efficacy in arrestin-3 recruitment might be due to an enhanced or stabilized binding of the peptide agonist to Y_4_R, as the affinity of arrestin to the Y_4_R remains unaffected by C1. In radioligand binding studies, we have further shown that C1 increases the affinity of the peptide ligands PP and PYY to Y_4_R membranes. We suggest that C1 helps to stabilize the agonist-bound receptor conformation via backbone and hydrophobic interactions with P^34^ of the orthosteric peptide ligand PP (Fig. 5D), and thus might increase the residence time in the ligand-bound state. The exact mechanism of C1 PAM activity in the potentiation of PP-mediated Y_4_R response, however, remains speculative. GPCRs are highly dynamic structures and can adopt multiple different conformational states during the transition from the inactive to the active state. It might be that binding of the PAM pre-forms the Y_4_R binding pocket for PP, thus facilitating agonist binding and G-protein association by lowering the energy barrier for the transition to the active state and stabilizing the extracellular ligand-binding domain. Binding of the PAM C1 might further modulate the allosteric interaction network within the TM bundle that links the extracellular ligand-binding pocket and the effector coupling interface, thereby altering the conformational dynamics of the Y_4_R. For the M2 muscarinic receptor (M2R), a modulator was shown to have positive allosteric effects on agonist affinity by stabilizing the extracellular domain of the receptor, mainly by changing the dynamics of the aromatic network linking the allosteric and orthosteric binding sites [72]. Another option is that a population of Y_4_R is already in the active state upon binding of C1. This is supported by data from GloSensor cAMP assay (Fig. 4A). Here, the compound C1 increases the basal Y_4_R activity in the absence of the peptide ligand by stabilizing the Y_4_R active conformation and shifting the equilibrium towards the active state. Furthermore, binding of the PAM might also allosterically stabilize the fully active Y_4_R-PP-G_i_ protein complex and reduce the oscillation between the active and inactive receptor state by increasing the residence time in the active state. This was recently shown by Cao et al. for a metabotropic glutamate 2 receptor (mGlu2R) PAM, in which the authors investigated the structural dynamics of mGlu2R in the presence and absence of the PAM [73]. However, to clarify the exact mechanism of C1 at Y_4_R, advanced experiments are needed. An interesting aspect might be to examine the dynamics of Y_4_R activation in the presence and/or absence of the PAM C1 and the NAM *(S)-*VU0637120. This might help to unveil different structural states of the Y_4_R and to better understand the mechanism of positive and negative allosteric modulation at Y_4_R.

Additionally, we demonstrated that C1 has a reduced allosteric effect for the low-affinity ligands NPY and PYY at Y_4_R. Sequence alignments of the NPY ligands (Fig. S3B) highlight the amino acid difference at position 34 of PP (P^34^) and NPY/PYY (Q^34^). As mentioned above, we assume that P^34^ of the orthosteric peptide ligand PP is engaged in hydrophobic interactions with C1 and thus strengthen the allosteric interaction between C1 and PP at Y_4_R. The sequence variability at position 34 appears to affect the orientation and interaction of NPY and PYY with C1 within the Y_4_R binding pocket, thereby reducing the allosteric cooperativity, which is indicated by the reduced allosteric modulation (2.2-fold EC_50_ shift for NPY, 2.9-fold EC_50_ shift for PYY versus 5-fold EC_50_ shift for PP).

Another important step towards understanding the mechanism of allosteric modulators involves gaining insights into their binding modes at GPCRs. The identification of allosteric binding sites often faces many challenges, primarily due to the diverse and unpredictable nature of these sites. Initially, we focused on the identification of Y_4_R domains that are relevant for the PAM activity of C1 by using Y_4_R/Y_1_R chimeras. Chimeric receptors are specifically used to identify amino acid residues differing between Y_4_R and Y_1_R, whereas identical residues are not covered by chimeras (Fig. 2A). For that reason, additional Y_4_R single mutants were selected, including residues that have previously been shown to be either important for the binding of the endogenous ligand PP [30] or the Y_4_R allosteric antagonist *(S)*-VU0637120 [35]. Notably, some of the Y_4_R residues identified to be relevant for the PAM C1, including T2.61, D6.59, and M7.43, have also been shown to be important for the interaction with the endogenous ligand PP. D6.59 is a highly conserved residue that is critical for all Y receptor subtypes to bind their NPY ligands [30, 31, 74]. As shown in the PP-Y_4_R cryo-EM structure, an ionic interaction between D6.59 and R^35^ of PP is formed [7]. As indicated by the docking model of C1 bound to Y_4_R (Fig. 8), D6.59 is not involved in direct interactions with the PAM, but seems to have a structural role in the stabilization of the PAM-bound active state structure. Furthermore, residue T2.61 has been proposed to be relevant for the interaction with C1 and PP. In the Y_4_R-PP-G_i1_ cryo-EM structure, T2.61 is involved in a polar interaction network with the distal amidated Y^36^ of PP. This residue is located deep inside the TM helical region at the bottom of the PP-Y_4_R binding pocket. We suggest that in the presence of the PAM, the Y_4_R adopts a different conformation, shifting the orientation of T2.61 more towards TM1, which might even tighten the binding of PP to Y_4_R. In this study, we have shown the high Y_4_R selectivity of C1. Comparison of the most relevant Y_4_R positions involved in C1 activity with Y_1_R, Y_2_R and Y_5_R revealed that most positions are unique at Y_4_R (Fig. S3A). This might strengthen the Y_4_R subtype-selective effect of C1 as interacting or stabilizing residues are not present at the other Y receptors.

**Fig. 10:**
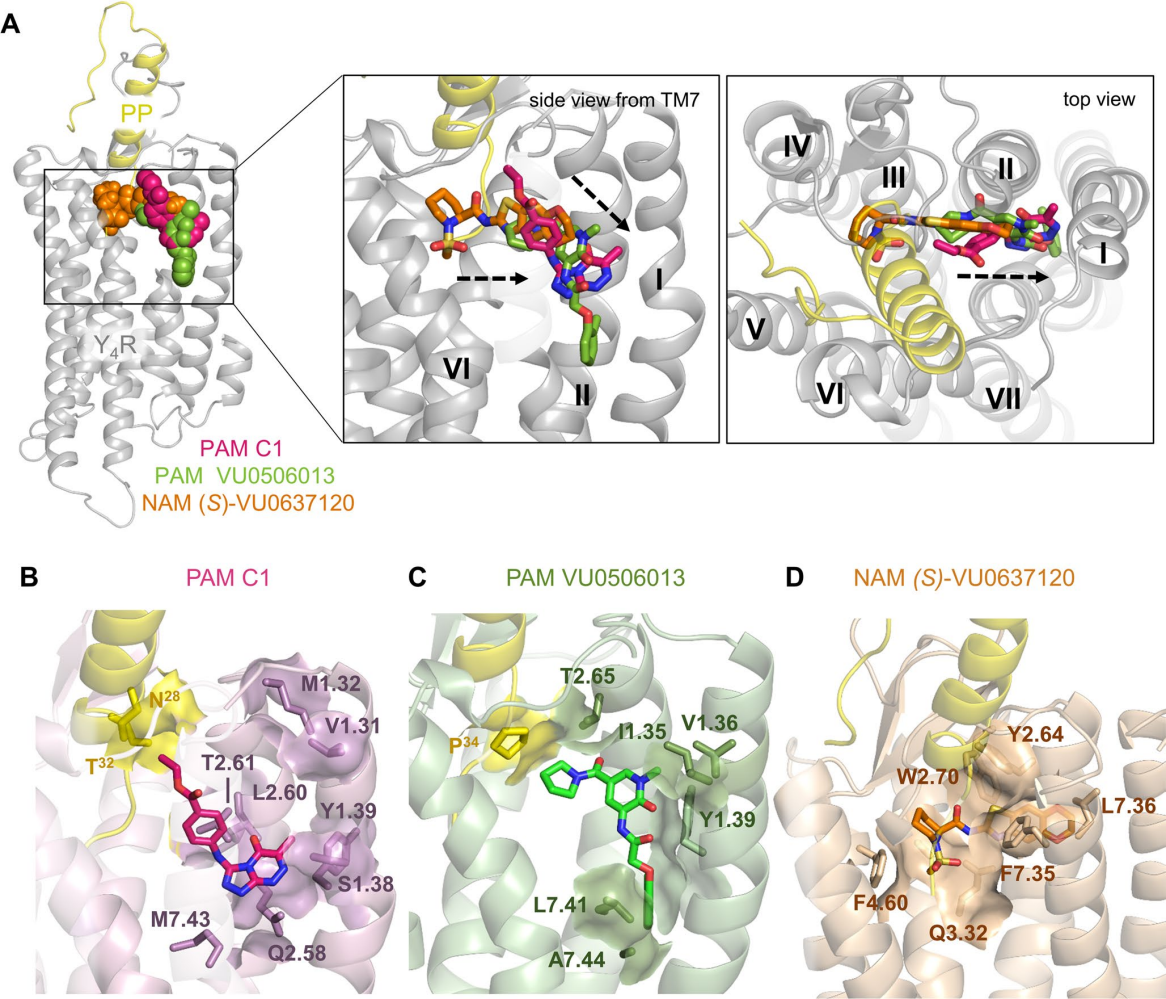
Comparison of overlapping allosteric and orthosteric ligand binding pockets at Y_4_R. (**A**) Overlay of the PAM C1 (cluster 2, pink sticks/spheres), PAM VU0506013 (green sticks/spheres), and NAM *(S)*-VU0637120 (orange sticks/spheres) binding sites at Y_4_R. Y_4_R and PP are shown in grey and yellow cartoon representation, respectively. As a template, the PP-Y_4_R cryo-EM structure was used (PDB: 7X9C). (**B–D**) Detailed binding mode of the pathway-independent PAM C1 (B), the PAM VU0506013 (C) and the allosteric antagonist (*S*)-VU0637120 (D) at Y_4_R. Relevant residues involved in the interaction with the small molecule are shown as sticks; the contact interface between the compounds and Y_4_R residues or PP (yellow) is shown in surface representation

As demonstrated, the PAM C1 interacts with an allosteric binding pocket at Y_4_R besides the C-terminal binding crevice of the agonist PP, and thus in a similar allosteric site already identified for the negative allosteric modulator *(S)-*VU0637120 and the G protein-preferring PAM VU0506013 (Fig. 10). However, these small molecules reveal significant structural and functional differences. The Y_4_R allosteric antagonist *(S)*-VU0637120 binds to an allosteric pocket in the Y_4_R TM core that slightly overlaps with the PP binding site and thus partially destabilizes PP binding through the interaction with PP-relevant residues, e.g. Y2.64, D2.68, Q3.32, F7.35 (Fig. 10D). Comparison of the *(S)-*VU0637120-bound Y_4_R model and the PP-C1-Y_4_R model further revealed clear differences in the conformation of the conserved rotamer toggle switch W6.48 and the nearby transmission switch F6.44 [75]. While these residues point inside the Y_4_R binding pocket in the *(S)*-VU0637120-bound conformation, the side chains of these two residues are flipped outward towards the intracellular surface in the PAM-bound state (Fig. S5), which is associated with an intracellular opening of TM6 and an enhanced interaction with the G protein. Comparison of the *(S)-*VU0637120 and C1 (cluster 2) binding pocket shows that C1 binds deeper within the TM helical bundle in proximity to the C-terminal binding groove of PP (Fig. 10) and stabilizes the interaction with the endogenous ligand through backbone interactions (N^28^/T^32^) and thus allosterically enhances its activity. In this study, we have shown that C1 is able to potentiate the G-protein signaling and arrestin-3 recruitment and acts pathway-independent towards the two major GPCR signaling pathways, the activation of G proteins and interactions with arrestin [1, 76, 77]. We assume that through the binding of C1, the Y_4_R-PP interaction is tightened, which results in an extended intracellular G-protein- and arrestin-coupling. In contrast to C1, the PAM VU0506013 shows a clear preference for modulating the G-protein pathway and has no effect on the binding affinity of the endogenous ligand PP [39]. VU0506013 binds to an allosteric pocket at Y_4_R located deeper inside the 7TM bundle that is more orientated towards TM1. Here, a clear difference in the activity of the two PAMs at the variants Y_4_(1.34–1.36_Y_1_) and Y_4_(T2.65A) has been identified. For C1, mutation of the residues 1.34–1.36 (Phe-Ile-Val) by the corresponding residues of Y_1_R (Thr-Leu-Ala) induced a *gain-of-function* activity, presumably by increasing the polar interaction interface with C1. In contrast, mutation of Y_4_(T2.65A) had no effect on C1 activity. For the previously described PAM VU0506013, both mutations led to a significant loss of PAM activity, indicating differences in the binding and mode of action of the small molecules even if they bind to similar pockets at Y_4_R. In contrast to C1, which mainly acts by allosterically tightening PP binding, the PAM VU0506013 might stabilize a G-protein favored receptor conformation and thus potentiates G-protein coupling. The identification of distinct Y_4_R allosteric pockets highlights that this receptor region can be precisely targeted by a very diverse set of ligands with different functional outcomes. The fine-tuning of a specific Y_4_R response by distinct small allosteric compounds forwards this receptor as an important pharmacological target in anti-obesity research.

## Conclusion

In conclusion, we identified a Y_4_R PAM, which significantly improves the allosteric cooperativity with the native peptide ligand PP and fine-tunes Y_4_R G-protein signaling in response to PP. Investigation of the close analog C2 revealed the structural importance of an ethyl acetate side chain for the high Y_4_R PAM activity and affinity. The identification of a structurally important moiety makes this an interesting scaffold and might further advance structure-activity relationship (SAR) studies. Assuming binding of C1 in cluster 2, removal of the ethyl acetate moiety might destabilize PP binding by the loss of the tight backbone interactions with PP. Additionally, we identified several residues within the extracellular region of Y_4_R in proximity to the endogenous ligand binding pocket that are important for C1 that might stabilize the PP-bound Y_4_R active state conformation. Taken together, this compound provides an interesting scaffold for further Y_4_R allosteric modulator research. For future work, it would be highly interesting to unravel the dynamics of Y_4_R activation in the presence of the PAM and the orthosteric agonist.

## Supplementary Information

Below is the link to the electronic supplementary material.

Supplementary Material 1 (1.20 MB)

## Data Availability

Data and materials are available upon request.

## Abbreviations

BRET: bioluminescence resonance energy transfer
COS-7: African green monkey kidney cells
CRC: concentration-response curve
DMEM: Dulbecco’s modified Eagle’s medium
DPBS: Dulbecco’s phosphate-buffered saline
ECL: extracellular loop
eYFP: enhanced yellow fluorescent protein
FBS: fetal bovine serum
HBSS: Hank’s balanced saline solution
HEK293: human embryonic kidney cells
ICL: intracellular loop
K_B_: equilibrium binding constant
M2R: M2 muscarinic receptor
mGlu2R: metabotropic glutamate 2 receptor
NPY: neuropeptide Y
PP: pancreatic polypeptide
PYY: peptide YY
RP-HPLC: reversed-phase high-performance liquid chromatography
SEM: standard error of the mean
TM: transmembrane
Y_1/2/4/5_R: neuropeptide Y receptor subtype 1/2/4/5
