# Supplementary material for: The pathway-independent positive allosteric modulator C1 allows for the identification of active Y4 receptor relevant positions
Source: Cell Mol Life Sci. 2026 Jan 13;83(1):74. doi: 10.1007/s00018-025-06019-7 (PMC12858701; doi:10.1007/s00018-025-06019-7)
Supplement: Supplementary file 1 — Supplementary Material 1 (1.20 MB) [file 18_2025_6019_MOESM1_ESM.pdf]

Supplementary Information

The pathway-independent positive allosteric modulator C1 allows for the identification of active Y_4 receptor relevant positions

Corinna Schüß[a*], Oanh Vu[b], Tim Pelczyk[a], Mario Schubert[a,c], Yu Du[d], Jan Stichel[a], C. David Weaver[d,e], Jens Meiler[b,f], Annette G. Beck-Sickinger[a]

[a] Institute of Biochemistry, Leipzig University, Leipzig 04103, Germany
[b] Department of Chemistry, Vanderbilt University, Nashville, TN 37235, United States
[c] Institute of Pharmacology and Toxicology, Technische Universität Dresden, Dresden 01307, Germany
[d] Department of Pharmacology, Vanderbilt University, Nashville, TN 37232, United States
[e] Institute of Chemical Biology, Vanderbilt University, Nashville, TN 37232, United States
[f] Institute for Drug Discovery, Leipzig University, Leipzig 04103, Germany

* Corresponding author
E-Mail: corinna.schuess@uni-leipzig.de

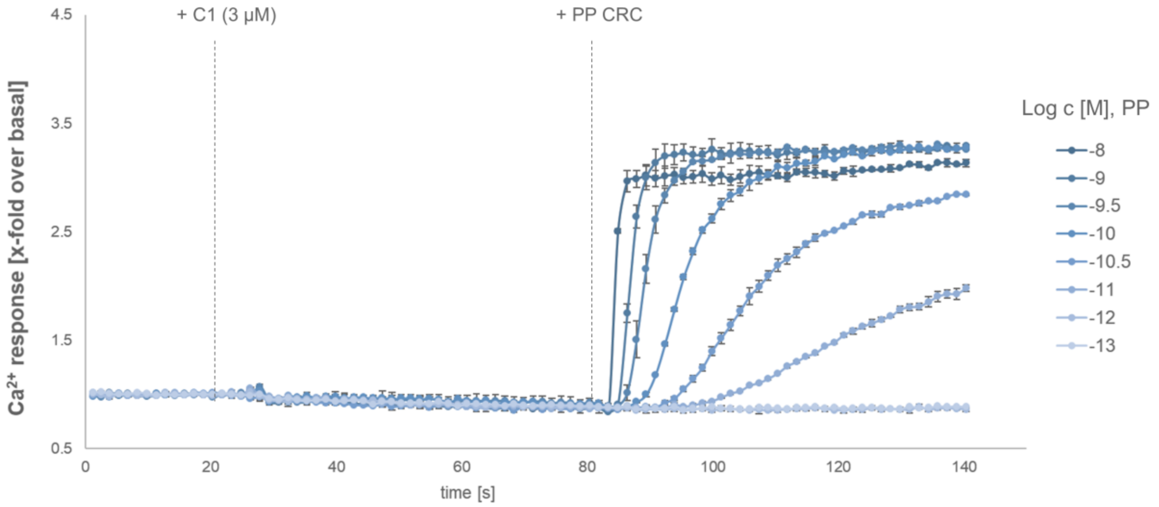

Fig. S1 C1 alone does not activate the Y$_4$R G-protein pathway. Ca^{2+} flux raw data are shown as *x*-fold over basal. After t = 20 s, C1 (3 µM) was added and Ca^{2+} levels were monitored for 60 s before Y$_4$R was stimulated with the agonist PP in a concentration-dependent manner (t = 80 s). Ca^{2+} response was measured over a total run time of 140 s. Ca^{2+} flux assay was performed in stably transfected COS-7_hY$_4$R-eYFP_Δ6G$\alpha_{qi4\text{-}myr}$ cells. Data represent the mean ± SEM from technical triplicates. Data show the Ca^{2+} flux raw traces from one representative out of N = 3 independent experiments.

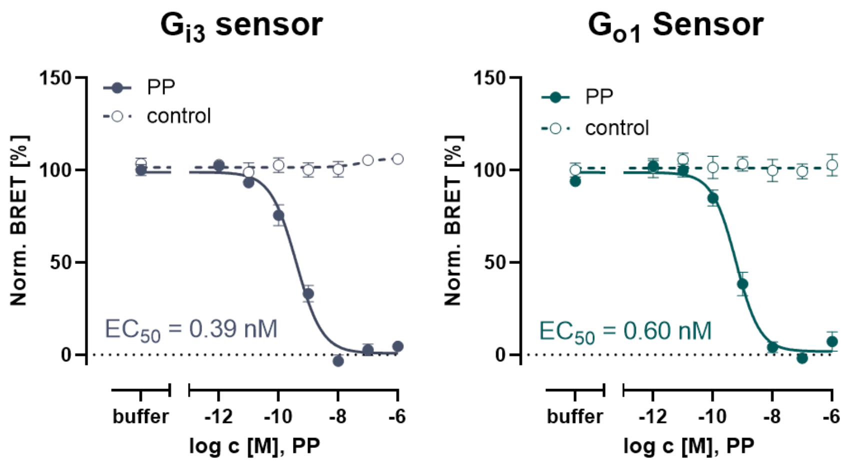

Fig S2. PP-dependent Y_4R G-protein activation using heterotrimeric G protein BRET sensors. Y_4R shows a ligand-dependent activation of heterotrimeric G protein sensors, while the mock control (pcDNA3) has no effect. G protein BRET with G_{i3} or G_{o1} BRET sensors was performed in COS-7 cells transiently expressing Y_4R or pcDNA3 mock control and G_{i3}/G_{o1} BRET sensor. Data are shown as mean ± SEM from at least four independent experiments, each performed in triplicates.

A

```
CLUSTAL O(1.2.4) multiple sequence alignment


sp|Q15761|NPY5R_HUMAN      ---MDLELDEYYNKT-LATENNTAATRNSDFPVW-----DDYKSSVDDLQYFLIGLYTFV  51
sp|P49146|NPY2R_HUMAN      MGPIGAEADENQTVEEMKVEQYGPQTTPRGELVPDPEPELIDSTKLIEVQVVLILAYCSI  60
sp|P25929|NPY1R_HUMAN      ---------MNST-LFSQVENHSVHSNFSEKNAQLLAFENDDCHLPLAMIFTLALAYGAV  50
sp|P50391|NPY4R_HUMAN      ---------MNTSHLLALLLPKSPQGENRSKPLGTPYNFSEHCQDSVDVMVFIVTSYSIE  51
                                       .                                   :   :   *

sp|Q15761|NPY5R_HUMAN      SLLGFMGNLLILMALMKKRNQKTTVNFLIGNLAFSDILVVLFCSPFTLTSVLLDQWMFGK  111
sp|P49146|NPY2R_HUMAN      ILLGVIGNSLVIHVVIKFKSMRTVTNFFIANLAVADLLVNTLCLPFTLTYTLMGEWKMGP  120
sp|P25929|NPY1R_HUMAN      IILGVSGNLALIIIILKQKEMRNVTNILIVNLSFSDLLVAIMCLPFTFVYTLMDHWVFGE  110
sp|P50391|NPY4R_HUMAN      TVVGVLGNLCLMCVTVRQKEKANVTNLLIANLAFSDFLMCLLCQPLTAVYTIMDYWIFGE  111
                            ::*. **  ::   :: :.  ...*::* **:.:*:*:  :* *:* . .::. * :*

sp|Q15761|NPY5R_HUMAN      VMCHIMPFLQCVSVLVSTLILISIAIVRYHMIKHPISNNLTANHGYFLIATVWTLGFAIC  171
sp|P49146|NPY2R_HUMAN      VLCHLVPYAQGLAVQVSTITLTVIALDRHRCIVYHLESKISKRISFLIIGLAWGISALLA  180
sp|P25929|NPY1R_HUMAN      AMCKLNPFVQCVSITVSIFSLVLIAVERHQLIINPRGWRPNNRHAYVGIAVIWVLAVASS  170
sp|P50391|NPY4R_HUMAN      TLCKMSAFIQCMSVTVSILSLVLVALERHQLIINPTGWKPSISQAYLGIVLIWVIACVLS  171
                           .:*::  : * ::: ** : *  :*: *:: *      . .   .:. *   * :.   .

sp|Q15761|NPY5R_HUMAN      SPLPVFHSLVELQET---FGSALLSSRYLCVESWPSDSYRI---AFTISLLLVQYILPLV  225
sp|P49146|NPY2R_HUMAN      SPLAIFREYSL-------IEIIPDFEIVACTEKWPGEEKSIYGTVYSLSSLLILYVLPLG  233
sp|P25929|NPY1R_HUMAN      LPFLIYQVMTDEPFQ--NVTLDAYKDKYVCFDQFPSDSHRL---SYTTLLLVLQYFGPLC  225
sp|P50391|NPY4R_HUMAN      LPFLANSILENVFHKNHSKALEFLADKVVCTESWPLAHHRT---IYTTFLLLFQYCLPLG  228
                            *:                      .   * :.:*          ::   *:. *  **

sp|Q15761|NPY5R_HUMAN      CLTVSHTSVCRSISCGLSNKENRLEENEMINLTLHPSKKSGPQVKLSGSHKWSYSFIKKH  285
sp|P49146|NPY2R_HUMAN      IISFSYTRIWSKLK--------------------------------------------NH  249
sp|P25929|NPY1R_HUMAN      FIFICYFKIYIRLK--------------------------------------------RR  241
sp|P50391|NPY4R_HUMAN      FILVCYARIYRCLQ--------------------------------------------RQ  244
                            : ..:  :   :.                                            .:

sp|Q15761|NPY5R_HUMAN      RRRYSKKTACVLPAPERPSQENHSRILPENFGSVRSQLSSSSKFIPGVPTCFEIKPEENS  345
sp|P49146|NPY2R_HUMAN      VSPGAAND----------------------------------------------------  257
sp|P25929|NPY1R_HUMAN      NNMMDKMR----------------------------------------------------  249
sp|P50391|NPY4R_HUMAN      GRVFHKG-----------------------------------------------------  251


sp|Q15761|NPY5R_HUMAN      DVHELRVKRSVTRIKKRSRSVFYRLTILILVFAVSWMPLHLFHVVTDFNDNLISNRHFKL  405
sp|P49146|NPY2R_HUMAN      -HYH-----------QRRQKTTKMLVCVVVVFAVSWLPLHAFQLAVDIDSQVLDLKEYKL  305
sp|P25929|NPY1R_HUMAN      -DNKYR--------SSETKRINIMLLSIVVAFAVCWLPLTIFNTVFDWNHQIIATCNHNL  300
sp|P50391|NPY4R_HUMAN      -TYSLR--------AGHMKQVNVVLVVMVVAFAVLWLPLHVFNSLEDWHHEAIPICHGNL  302
                                           . :     *  :::.*** *:**  *:   * . : :   . :*

sp|Q15761|NPY5R_HUMAN      VYCICHLLGMMSCCLNPILYGFLNNGIKADLVSLIH-CLHM-------------------  445
sp|P49146|NPY2R_HUMAN      IFTVFHIIAMCSTFANPLLYGWMNSNYRKAFLSAFR-CEQRLD----------AIHSEVS  354
sp|P25929|NPY1R_HUMAN      LFLLCHLTAMISTCVNPIFYGFLNKNFQRDLQFFFNFCDFRSRDDDYETIAMSTMHTDVS  360
sp|P50391|NPY4R_HUMAN      IFLVCHLLAMASTCVNPFIYGFLNTNFKKEIKALVLTCQQSAPLEESEHLPLSTVHTEVS  362
                           :: : *: .* *   **::**::*.. :  :   .  *

sp|Q15761|NPY5R_HUMAN      -----------------------------   445
sp|P49146|NPY2R_HUMAN      VTFKAKKNLEVRKNSGPNDSFTEATNV--   381
sp|P25929|NPY1R_HUMAN      KT-----SLKQASPVAFKKINNNDDNEKI   384
sp|P50391|NPY4R_HUMAN      KG-----SLRLSGRSNPI-----------   375
```

B **Sequence alignment NPY peptides**

```
          1          11         21         31
PP        APLEPVYPGD NATPEQMAQY AADLRRYINM LTRPRY-NH2
PYY       YPIKPEAPGE DASPEELNRY YASLRHYLNL VTRQRY-NH2
NPY       YPSKPDNPGE DAPAEDMARY YSALRHYINL ITRQRY-NH2
```

Fig. S3 Sequence alignment of human Y receptors and NPY ligands. (**A**) The receptor sequence alignment of human Y_1R, Y_2R, Y_4R and Y_5R was created using Clustal O(1.2.4.) multiple sequence alignment [1]. Red color represents important Y_4R positions relevant for C1 activity that differ between Y receptors, while blue marks positions that are more conserved. (**B**) Sequence alignment of NPY receptor ligands PP, PYY, and NPY. Position 34 of the ligands is highlighted in red.

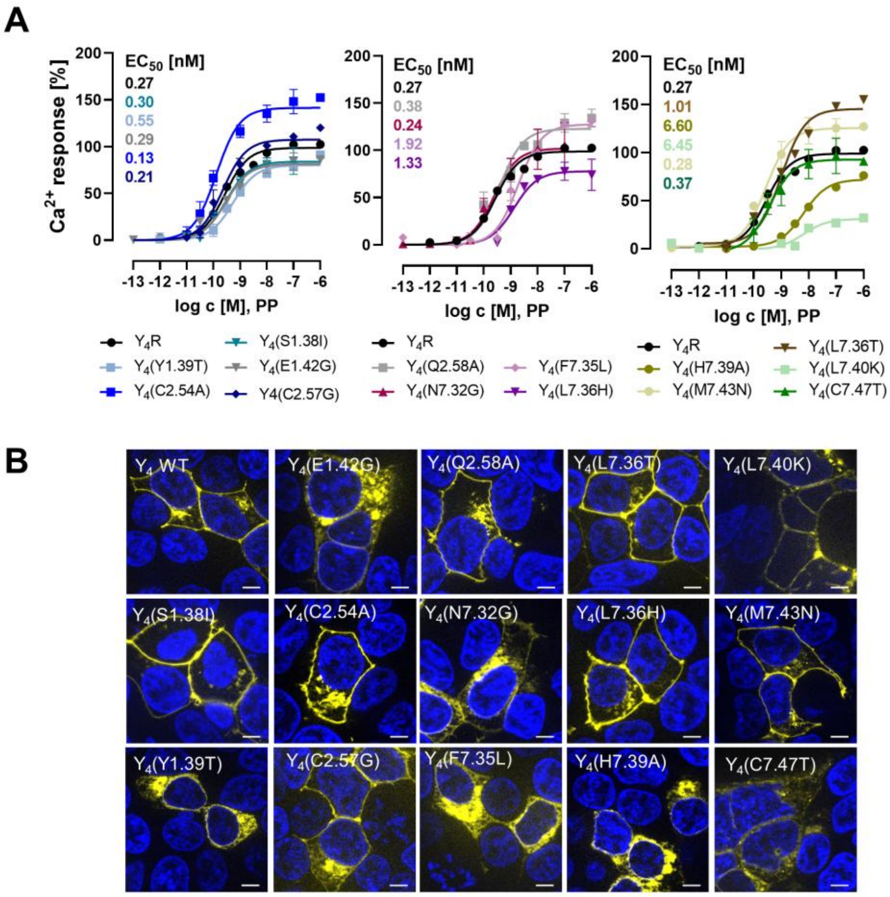

Fig. S4 Characterization of new Y_4R variants. (**A**) PP activation of Y_4R and variants was measured using Ca^{2+} flux assay in COS-7 cells transiently expressing one receptor-eYFP variant and the chimeric G protein $\Delta 6G\alpha_{qi4\text{-}myr}$. Data are shown as mean ± SEM from N ≥ 2 independent experiments. (**B**) Membrane expression of Y_4R variants, C-terminally fused to eYFP (yellow), was studied by live cell fluorescence microscopy of transiently transfected HEK293 cells. Cell nuclei were stained with Hoechst33342 (blue). Scale bar is 10 µm. Pictures are representatives from N ≥ 2 independent experiments.

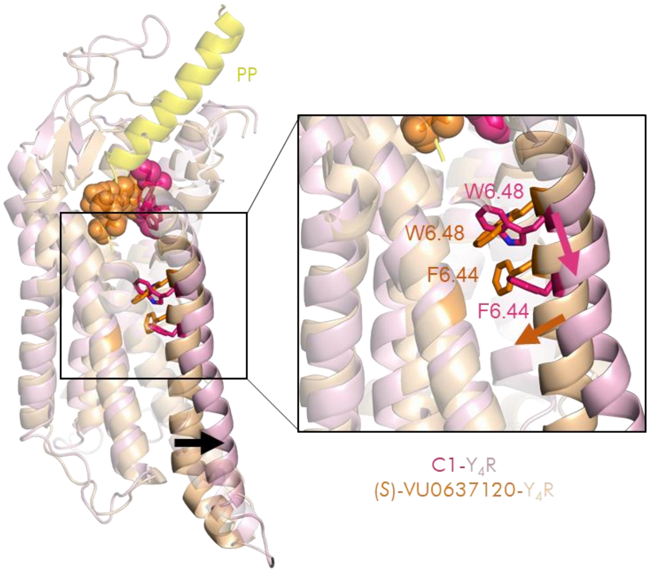

Fig. S5 Comparison of the rotamer toggle and transmission switch and TM6 movement in the active PP-C1-Y_4R model and the inactive *(S)*-VU0637120-Y_4R docking pose. In the PAM-bound Y_4R state, the rotamer switch W6.48 and the nearby transmission switch F6.44 are flipped and point away from the central axis, associated with the intracellular opening of TM6 for G-protein coupling. The C1- and *(S)*-VU0637120-Y_4R structures are shown in orange and pale pink cartoon representation, respectively, PP is shown as yellow cartoon. The compounds C1 and *(S)*-VU0637120 are highlighted as pink and yellow spheres. The W6.48 rotamer switch and F6.44 transmission switch are represented as sticks.

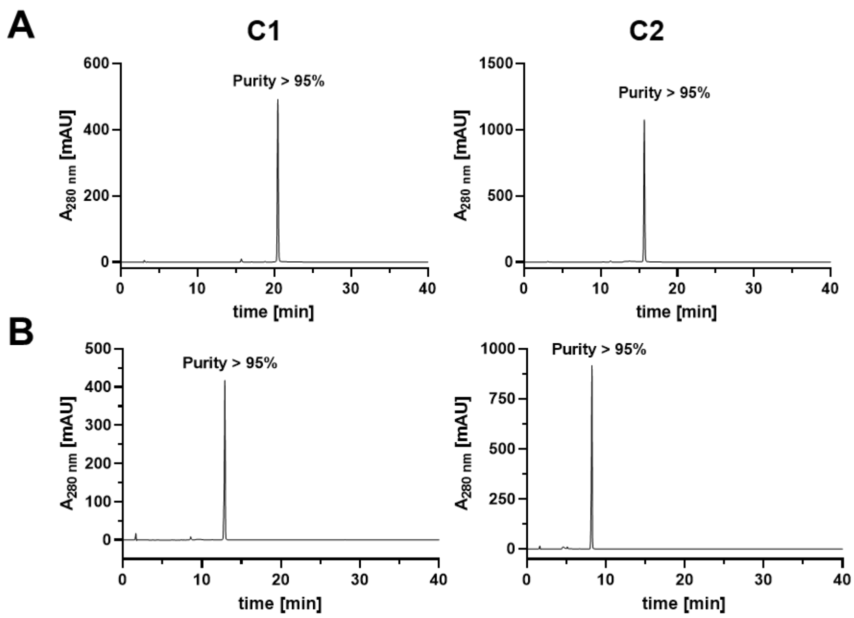

Fig. S6 RP-HPLC traces of C1 and C2 to confirm homogeneity. (**A**) HPLC chromatogram of C1 and C2 at 280 nm using a Jupiter Proteo C12 column (Phenomenex: 250.0 mm x 4.6 mm, 4.0 µm; 90 Å) with a linear gradient of 20–70% (*v*/*v*) eluent B (ACN with 0.08% TFA (*v*/*v*)) in eluent A (water with 0.1% TFA (*v*/*v*)) over 40 min at a flow rate of 1 mL/min, 40°C. (**B**) HPLC chromatogram of C1 and C2 at 280 nm using an Aeris Peptide XB-C18 column (Phenomenex: 250.0 mm x 4.6 mm, 3.6 µm; 100 Å) with a linear gradient of 20–70% (*v*/*v*) eluent B (ACN with 0.08% TFA (*v*/*v*)) in eluent A (water with 0.1% TFA (*v*/*v*)) over 40 min at a flow rate of 1.55 mL/min, 40°C.

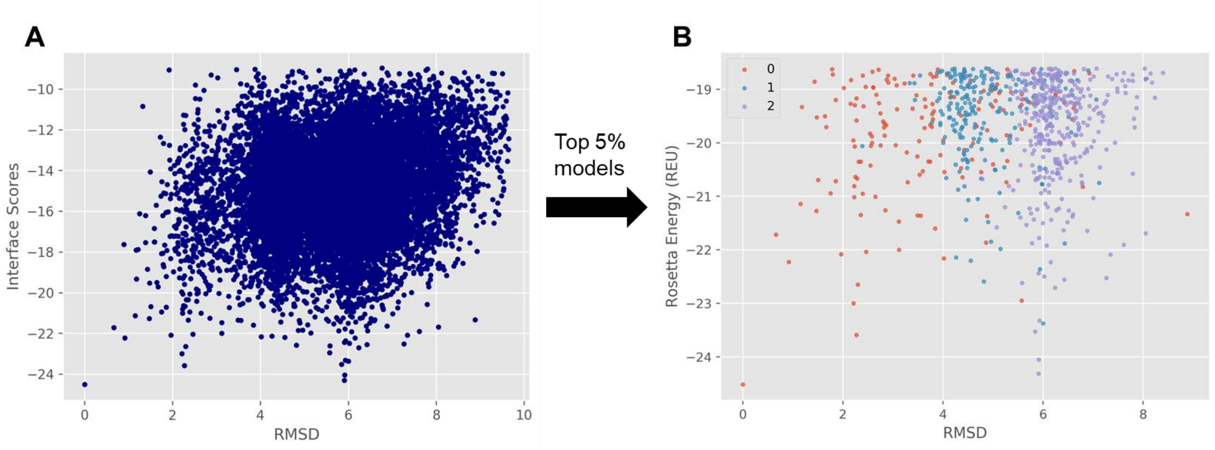

Fig. S7 The Rosetta interface scores versus ligand RMSDs of the output models (**A**) and top 5% output models (**B**) from the final round of induced fit docking. The top 5% models were selected based on the C1-Y_4R interface score, and were clustered into three clusters. The representative binding pose of cluster 2 was chosen as the final predicted docking model.

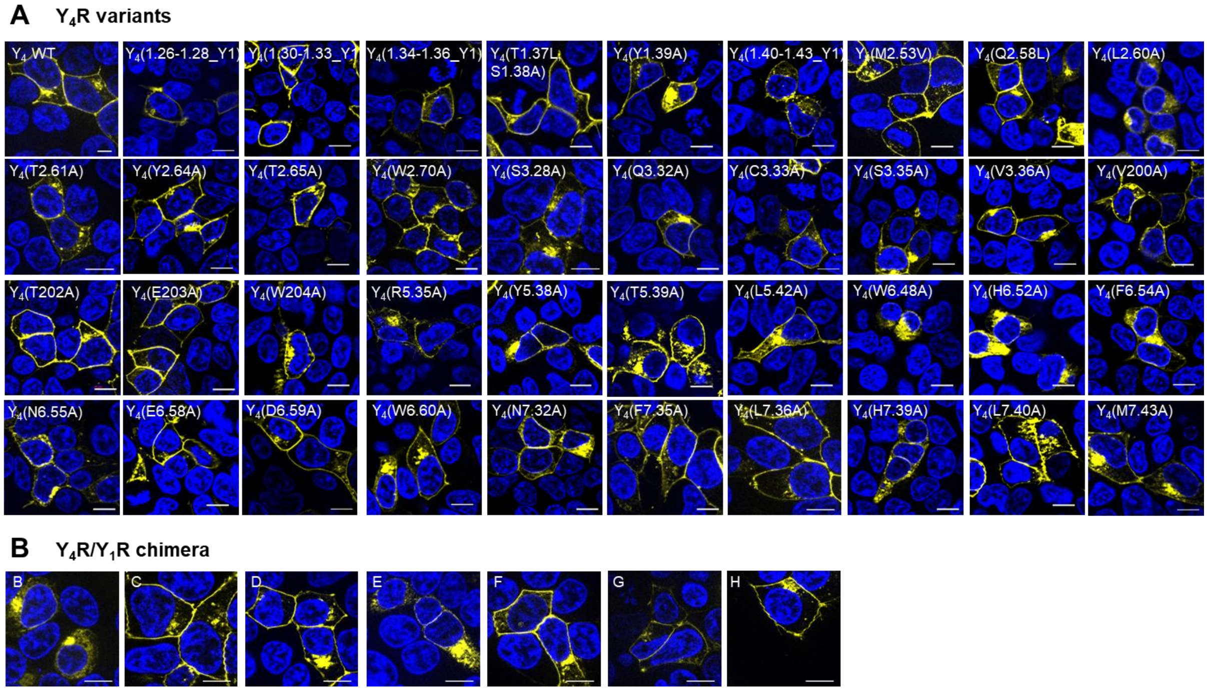

Fig. S8 Membrane expression of already published Y_4R variants (**A**) and Y_4R/Y_1R chimera (**B**), C-terminally fused to eYFP (yellow), was studied by live cell fluorescence microscopy in transiently transfected HEK293 cells. Cell nuclei were stained with Hoechst33342 (blue). Scale bar is 10 µm. Pictures are representatives from N ≥ 2 independent experiments.

Table S1 Mutagenesis data from C1. Y_4R/Y_1R chimeras and Y_4R single variants with a clear loss in potentiation are highlighted in grey. Y_4R/Y_1R chimeras and Y_4R variants were investigated by Ca^{2+} flux assay in transiently transfected COS-7 cells.

Receptor construct	Potentiation 10 µM of C1 [%] ± SEM [a]	N	Potentiation 30 µM of C1 [%] ± SEM [a]	N	Transfection efficacy [%] ± SEM [b]
			**Y_4R/Y_1R chimeras**		
Y_4R WT	22.7 ± 1.8	24	25.0 ± 2.6	23	100 ± 5
Y_4(N-TM5E_Y_1)	5.7 ± 5.9	4	11.3 ± 4.8	3	43 ± 41
Y_4(N-TM1E_Y_1)	-5.9 ± 6.9	4	5.7 ± 7.4	3	103 ± 10
Y_4(ICL1, TM2_Y_1)	10.9 ± 1.9	4	9.4 ± 0.3	3	105 ± 8
Y_1(N-TM5E_Y_4)	10.9 ± 7.7	5	11.1 ± 10.4	4	79 ± 8
Y_4(ECL1_Y_1)	2.6 ± 5.1	5	6.2 ± 8.9	4	42 ± 18
Y_4(ECL2_Y_1)	22.0 ± 7.8	5	26.1 ± 8.8	4	75 ± 7
Y_4(ECL3_Y_1)	17.0 ± 2.8	5	26.3 ± 2.0	4	67 ± 16
			**Y_4R variants**		
Y_4(1.26-1.28_Y_1)	6.9 ± 2.2	4	5.2 ± 3.3	4	96 ± 6
Y_4(1.30-1.33_Y_1)	6.9 ± 4.9	6	11.4 ± 7.1	6	68 ± 10
Y_4(1.34-1.36_Y_1)	38.4 ± 3.9	4	37.0 ± 4.1	4	64 ± 9
Y_4(T1.37L, S1.38A)	8.3 ± 5.6	4	10.7 ± 1.5	4	111 ± 15
Y_4(Y1.39A)	6.4 ± 2.4	3	9.1 ± 1.5	3	79 ± 7
Y_4(1.40-1.43_Y_1)	6.2 ± 1.6	4	13.2 ± 3.9	4	103 ± 7
Y_4(M2.53V)	15.0 ± 5.5	4	19.7 ± 4.4	4	87 ± 22
Y_4(Q2.58L)	8.1 ± 4.3	6	8.1 ± 3.8	6	103 ± 14
Y_4(L2.60A)	6.4 ± 4.9	4	7.0 ± 7.7	4	72 ± 17
Y_4(T2.61A)	6.2 ± 2.2	4	9.9 ± 2.0	4	67 ± 13
Y_4(Y2.64A)	10.4 ± 4.0	4	13.1 ± 3.6	4	51 ± 13
Y_4(Y2.64F)	16.4 ± 1.7	3	14.7 ± 5.0	3	123 ± 25
Y_4(T2.65A)	18.4 ± 2.1	4	23.8 ± 4.7	4	90 ± 7
Y_4(W2.70A)	16.2 ± 5.2	5	15.9 ± 5.1	5	79 ± 8
Y_4(S3.28A)	10.7 ± 5.0	5	9.9 ± 4.2	5	103 ± 8
Y_4(Q3.32A)	15.4 ± 8.3	5	20.3 ± 6.8	5	90 ± 4
Y_4(C3.33A)	8.4 ± 4.1	3	11.1 ± 2.6	3	86 ± 19
Y_4(S3.35A)	11.4 ± 7.3	3	21.0 ± 9.5	3	69 ± 9
Y_4(V3.36A)	16.5 ± 7.0	3	15.7 ± 5.2	3	86 ± 16
Y_4(V5.24A)	14.5 ± 0.8	3	12.0 ± 5.9	3	111 ± 15
Y_4(T5.26A)	11.7 ± 4.0	4	16.0 ± 3.1	4	97 ± 15
Y_4(E5.27A)	12.1 ± 5.2	4	11.6 ± 4.8	4	116 ± 7
Y_4(W5.29A)	4.0 ± 4.7	4	12.0 ± 6.4	4	99 ± 7
Y_4(R5.35A)	9.4 ± 3.6	4	15.0 ± 3.6	4	88 ± 5
Y_4(Y5.38A)	9.3 ± 2.6	3	11.8 ± 3.7	3	68 ± 6-
Y_4(T5.39A)	12.8 ± 2.0	4	22.7 ± 3.9	4	81 ± 5
Y_4(L5.42A)	10.1 ± 4.3	3	8.6 ± 9.8	3	68 ± 13
Y_4(W6.48A)	25.9 ± 12.0	3	24.3 ± 12.5	3	79 ± 7
Y_4(H6.52A)	9.7 ± 4.3	3	8.6 ± 1.2	2	92 ± 6
Y_4(F6.54A)	13.5 ± 5.5	3	13.9 ± 7.0	2	95 ± 5
Y_4(N6.55A)	12.1 ± 4.0	3	18.7 ± 7.3	2	108 ± 5
Y_4(E6.58A)	30.8 ±11.2	4	24.9 ± 7.1	3	100 ± 9
Y_4(D6.59A)	2.9 ± 2.4	6	8.2 ± 2.0	5	106 ± 6
Y_4(W6.60A)	8.2 ± 2.2	5	6.8 ± 2.3	4	79 ± 7
Y_4(N7.32A)	13.3 ± 6.7	2	19.1 ± 6.6	5	72 ± 17
Y_4(F7.35A)	10.4 ± 6.4	3	13.4 ± 4.7	3	73 ± 23
Y_4(L7.36A)	11.2 ± 6.9	4	24.9 ± 10.2	4	72 ± 17
Y_4(H7.39A)	16.5 ± 0.4	3	17.0 ± 3.2	3	72 ± 14
Y_4(H7.39P)	11.4 ± 3.8	3	10.5 ± 4.5	3	130 ± 4
Y_4(L7.40A)	16.5 ± 8.1	4	19.8 ± 6.8	4	23 ± 4
Y_4(M7.43A)	5.5 ± 2.6	3	6.1 ± 1.4	3	69 ± 8

[a] [%] Potentiation was determined as the absolute potentiation of a PP EC_{30} response by C1 relative to the DMSO control.

[b] Transfection efficacy was determined by measuring eYFP fluorescence from transiently transfected receptor-eYFP constructs. Transfection efficacy was normalized to Y_4R WT.

Table S2 Per residue binding energy and mutagenesis agreement scores for the three docking clusters.

res#	resid	cluster0	cluster1	cluster2
2	1.26			
3	1.27			
4	1.28			
6	1.30			
7	1.31			
8	1.32			
9	1.33			
13	1.37	-0.05	-0.02	-0.14
14	1.38	-0.44	-0.42	-2.77
15	1.39	-1.45	-0.86	-1.12
16	1.40	-0.10	0.00	-0.03
17	1.41	-0.03		
18	1.42	-0.49	-0.25	-1.52
19	1.43	-0.81	-0.55	-0.62
62	2.58	-2.59	-2.97	-3.38
64	2.60	-0.08	-0.20	-0.34
65	2.61	-1.82	-2.11	-1.81
68	2.64			
69	2.65	-1.24	-0.34	-1.20
84	3.28		-0.41	
88	3.32	-0.45	-2.18	-0.93
89	3.33		-0.02	
91	3.35			
178	5.35			
181	5.38			
182	5.39			
185	5.42			
248	6.52	-0.18	-0.27	-0.19
250	6.54			
251	6.55			
255	6.59			
256	6.60			
267	7.32			
270	7.35			
275	7.40	-2.99	-3.43	-3.38
278	7.43		-0.30	-0.21

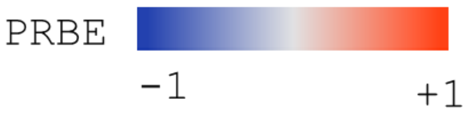

	cluster0	cluster1	cluster2
Score	12.73	14.32	17.65

Supplementary Method

Cell surface expression

Membrane expression of Y_4R variants was investigated by live cell fluorescence microscopy. Therefore, 120,000 HEK293 cells were seeded into poly-D-Lys coated (0.0001%) µ-slide eight-well chambers (IBIDI) and incubated overnight under standard cell culture conditions. At a density of about 80%, cells were transfected with 1 µg plasmid cDNA of Y_4R or variants, C-terminally labeled with eYFP, and 1 µL of Lipofectamine2000 transfection reagent, as described [2]. After 18 h of incubation under standard conditions, cells were starved with 200 µL of OptiMEM and cell nuclei were stained with Hoechst33342 for 30 min at 37 °C. The staining solution was removed and replaced by pure OptiMEM. Fluorescence microscopy was performed using an ApoTome.2 imaging system, with an AxioVert ObserverZ1 fluorescence microscope (Zeiss). Hoechst33342 was imaged by a DAPI filter (filter set 49, excitation G 365, emission BP 445/50), eYFP was imaged by a YFP filter (filter set 46, excitation BP 500/20, BP 535/30) with individual exposure times.

Supplementary References

1. Sievers F, Wilm A, Dineen D, Gibson TJ, Karplus K, Li W, Lopez R, McWilliam H, Remmert M, Söding J, Thompson JD, Higgins DG (2011) Fast, scalable generation of high-quality protein multiple sequence alignments using Clustal Omega. Mol. Syst. Biol. 7:539. https://doi.org/10.1038/msb.2011.75
2. Rathmann D, Pedragosa-Badia X, Beck-Sickinger AG (2013) In vitro modification of substituted cysteines as tool to study receptor functionality and structure-activity relationships. Anal. Biochem. 439:173–183. https://doi.org/10.1016/j.ab.2013.04.015
